# H_2_S Increases Blood Pressure via Activation of L‐Type Calcium Channels with Mediation by HS^•^ Generated from Reactions with Oxyhemoglobin

**DOI:** 10.1002/advs.202305866

**Published:** 2024-04-29

**Authors:** Taiming Liu, Meijuan Zhang, Shawn Hanson, Rucha Juarez, Sean Wilson, Hobe Schroeder, Qian Li, Lingchao Zhu, Guangyu Zhang, Arlin B. Blood

**Affiliations:** ^1^ Division of Neonatology Department of Pediatrics Loma Linda University School of Medicine Loma Linda CA 92354 USA; ^2^ Lawrence D. Longo Center for Perinatal Biology Loma Linda University School of Medicine Loma Linda CA 92354 USA; ^3^ Department of Medicine Gregory Fleming James Cystic Fibrosis Research Center University of Alabama at Birmingham Birmingham AL 35294 UK; ^4^ Department of Chemistry University of California Riverside CA 92521 USA; ^5^ Mass spectrometry core facility Loma Linda University Loma Linda CA 92354 USA

**Keywords:** blood pressure, hemoglobin, hydrogen sulfide, L‐type calcium channel, thiyl radical, vasoconstriction

## Abstract

Although the gasotransmitter hydrogen sulfide (H_2_S) is well known for its vasodilatory effects, H_2_S also exhibits vasoconstricting properties. Herein, it is demonstrated that administration of H_2_S as intravenous sodium sulfide (Na_2_S) increased blood pressure in sheep and rats, and this effect persisted after H_2_S has disappeared from the blood. Inhibition of the L‐type calcium channel (LTCC) diminished the hypertensive effects. Incubation of Na_2_S with whole blood, red blood cells, methemoglobin, or oxyhemoglobin produced a hypertensive product of H_2_S, which is not hydrogen thioperoxide, metHb‐SH^−^ complexes, per‐/poly‐ sulfides, or thiolsulfate, but rather a labile intermediate. One‐electron oxidation of H_2_S by oxyhemoglobin generated its redox cousin, sulfhydryl radical (HS**^•^)**. Consistent with the role of HS**^•^** as the hypertensive intermediate, scavenging HS**^•^** inhibited Na_2_S‐induced vasoconstriction and activation of LTCCs. In conclusion, H_2_S causes vasoconstriction that is dependent on the activation of LTCCs and generation of HS**^•^** by oxyhemoglobin.

## Graphical abstract

H_2_S, a vasodilatory gasotransmitter, is thought to be primarily metabolized in mitochondria. This work describes a novel function of oxyhemoglobin whereby it rapidly metabolizes the vasodilatory H_2_S into its redox cousin, the vasoconstrictive HS**^•^**. This study not only suggests blood is a major sink of H_2_S, but also opens the door for investigating the radical pathways of H_2_S signaling.

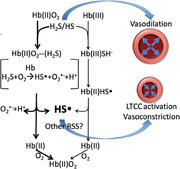

## 1 Introduction

Hydrogen sulfide (H_2_S) is an endogenous gasotransmitter with diverse physiological and pathological functions.^[^ ^1^ ^]^ Although H_2_S is best known as a vasodilator, it has been frequently demonstrated to induce vasoconstriction.^[^ ^2^, ^3^ ^]^ The vasoactivity of H_2_S has been shown to vary according to animal species, organ type, and experimental conditions, such as oxygen tensions, concentration of H_2_S, buffer composition, and reagents used for vessel preconstriction.^[^ ^4^ ^]^ For instance, H_2_S generally induces vasoconstriction at concentrations below 100 µm but causes vasodilation at higher concentrations.^[^ ^5^, ^6^, ^7^, ^8^ ^]^ Although many pathways have been suggested to explain these opposing vasoactive responses to H_2_S,^[^ ^9^ ^]^ the underlying mechanism that determines which response will occur remains unclear.

Although the vasoactivity of H_2_S in isolated arteries has been thoroughly researched, few studies have been carried out in vivo; thus, key pieces of knowledge remain unknown, considering that H_2_S diffuses freely and tends to be oxidized.^[^ ^10^ ^]^ H_2_S has been shown to disappear rapidly from the blood of various species in vivo and in vitro.^[^ ^11^, ^12^, ^13^ ^]^ However, the mechanism driving the metabolism of H_2_S in blood remains unclear. Studies have suggested that the direct reaction of H_2_S with O_2_ is too slow for biological relevance,^[^ ^14^, ^15^ ^]^ and the well‐known reaction between H_2_S and oxyhemoglobin (HbO_2_) to form sulfhemoglobin‐ (sulfHb) also seems inefficient.^[^ ^16^ ^]^ It has been recently suggested that methemoglobin (metHb) can serve as a site of H_2_S oxidation that generates persulfide (R_1_SSR_2_), polysulfide (R_1_S_n_R_2_), and thiosulfate (S_2_O_3_^2−^), possibly via a sulfhydryl radical (HS**^•^)** intermediate.^[^ ^16^, ^17^, ^18^ ^]^ O_2_ has been suggested to play a critical role in the intracellular metabolism of H_2_S via the functions of a number of mitochondrial enzymes.^[^ ^19^ ^]^ However, a role for O_2_ in H_2_S metabolism in blood has not been identified thus far.^[^ ^17^, ^20^ ^]^

In this study, we demonstrate that H_2_S, administered as sodium sulfide (Na_2_S), is hypertensive in sheep and rats. In addition, this effect persists after the injected H_2_S has disappeared from blood, suggesting the presence of a hypertensive metabolite. To identify this hypertensive metabolite, we examined the vasoactivity and reaction products generated by the mixture of Na_2_S and blood components. Using electron paramagnetic resonance, we found evidence for the production of HS**^•^** through single electron oxidation of H_2_S by HbO_2_. Thus, the role of HS**^•^** in the vasoconstrictive effects of H_2_S and the involvement of the L‐type calcium channel (LTCC) were examined in vivo and in vitro using ascorbate as an HS**^•^** scavenger.

## 2 Results

### 2.1 Hypertensive Effects and Kinetics of H_2_S

**Figure 1:**
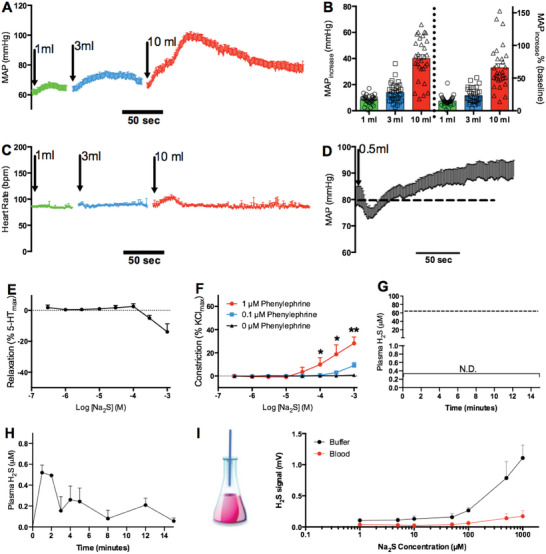
Hypertensive effects and pharmacokinetics of H_2_S. A) Averaged traces from 32 sheep showing a dose‐dependent increase in mean arterial blood pressure (MAP) by Na_2_S (intravenous bolus injection of 128 mm). B) Absolute (left) and relative (right) increase of MAP in (A). C) Averaged traces of heart rate responses in (A). D) Na_2_S (intravenous bolus injection of 12.8 mm) transiently decreased and then raised MAP in rats. *n* = 5. E,F) Wire myography. E) Na_2_S did not relax 5‐HT preconstricted sheep mesenteric arteries at concentrations lower than 100 µm. *n* = 5. F) Na_2_S was not vasoconstrictive when applied alone but produced vasoconstriction in the presence of phenylephrine at increasing concentrations (*n* = 5). After phenylephrine was added, tension was tuned to baseline before the addition of Na_2_S. Paired t‐test. G) No H_2_S was detected in vivo after injection of 10 mL of 128 mm Na_2_S, which is estimated to result in 65 µm H_2_S (18.6% of total 350 µm H_2_S/HS^−^/S^2−^ at pH 7.4) in blood, as marked by the dashed line. N.D.:not detectable with a detection limit of 100 nm. H) Pharmacokinetics of H_2_S in vitro after addition of a starting concentration of 100 µm of Na_2_S into blood. *n* = 5. I) Headspace detection of H_2_S with an H_2_S‐specific amperometric electrode. *n* = 10.

Intravenous administration of Na_2_S to ewes dose‐dependently increased their mean arterial blood pressure (MAP) as well as their systolic and diastolic pressures, while their heart rate was largely unaltered (Figure **1**A–C; Figure S2a,b, Supporting Information). In rats, Na_2_S administration resulted in a transient decrease followed by a prolonged increase in MAP, further supporting the previously reported hypertensive effects of H_2_S ^[^ ^21^ ^]^ (Figure 1D).

The vasoactivity of H_2_S was also tested in isolated sheep arteries using wire myography. Relaxation was not observed in response to Na_2_S at concentrations up to 100 µm in either preconstricted femoral (Figure S3, Supporting Information) or mesenteric (Figure 1E) arteries. Mesenteric arteries were selected as representatives of peripheral arteries to study the contractile effects of H_2_S. Although Na_2_S is not vasoactive when applied alone, dose‐dependent contraction of mesenteric arteries was observed when Na_2_S was applied in the presence of 0.1 or 1 µM phenylephrine. The Na_2_S‐induced contraction was stronger at 1 µm phenylephrine than at 0.1 µm phenylephrine (Figure 1F). Notably, phenylephrine alone only stimulated detectable contraction at or above 1 µm but not at 0.1 µm (Figure S4, Supporting Information).

Pharmacokinetic studies of H_2_S in blood samples collected from ewes following intravenous injections of Na_2_S demonstrated that H_2_S had vanished (<100 nM; not detectable) within one minute (Figure 1G). Similarly, H_2_S was below 1 µm within 1 minute in fresh plasma samples when Na_2_S was added to sheep blood at an initial concentration of 100 µm, suggesting that H_2_S is cleared extremely rapidly by blood (Figure 1H). In addition, headspace detection of H_2_S by a H_2_S‐specific amperometric electrode demonstrated that little H_2_S could escape from blood after up to 1 mm Na_2_S was added (Figure 1I).

### 2.2 Mechanisms for the Hypertensive Effects of H_2_S

Carotid chemoreceptors, ganglionic activity, alpha‐1 adrenergic activity, prostaglandins, the cAMP pathway, and K_ATP_ channels are important factors in blood pressure regulation, and some have been proposed to play a role in the effects of H_2_S.^[^ ^3^ ^]^ We initially examined all of these pathways but obtained null results, as presented in Figure S5 (Supporting Information). Specifically, denervation of the carotid body (Figure S5a,b, Supporting Information) did not significantly (p = 0.2158 versus Control in Figure 1A,B) alter the maximal hypertensive effects of H_2_S. Similarly, the ganglionic activity blocker hexamethonium (*p* = 0.4427), alpha‐1 adrenergic receptor blocker prazosin (*p* = 0.2322), COX1/2 inhibitor indomethacin (*p* = 0.2049), cAMP pathway agonist isoproterenol (*p* = 0.1144), and K_ATP_ channel blocker glibenclamide (*p* = 0.9718) also failed to significantly alter the maximal hypertensive effects of H_2_S (Figure S5c‐l, Supporting Information), suggesting that these pathways are not likely involved in the hypertensive mechanism.

### 2.3 Interaction with the NO‐cGMP Pathway and a Role of LTCC

**Figure 2:**
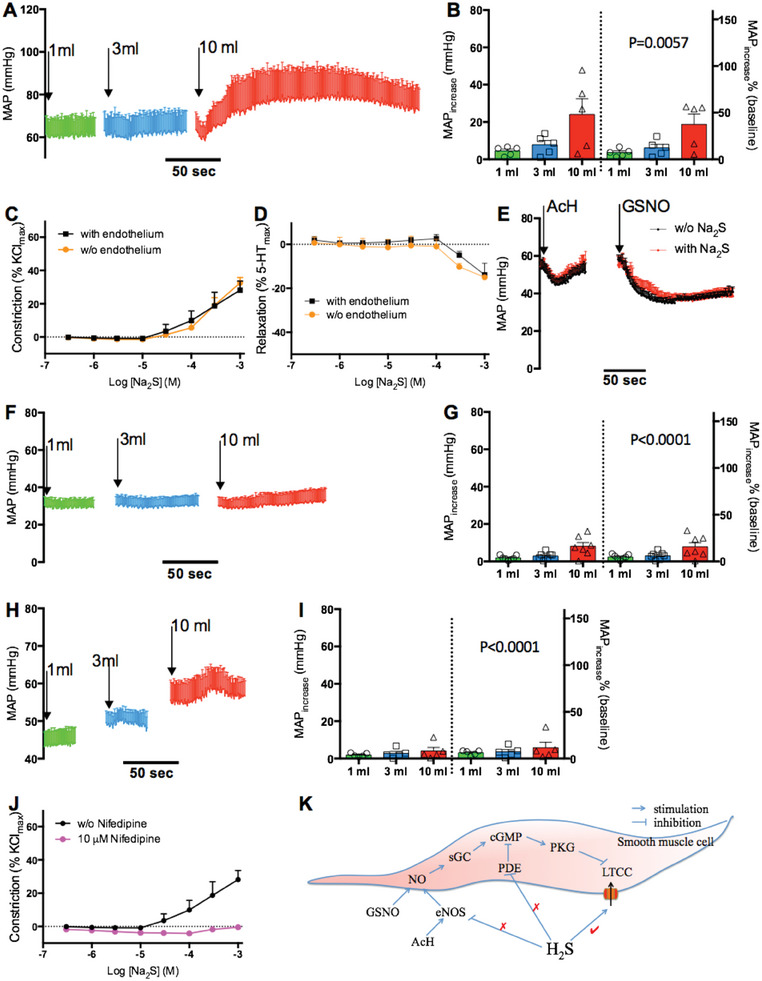
Interaction with the NO‐cGMP pathway and the role of L‐type calcium channel (LTCC) in the vasoconstricting effects of H_2_S. A,B) The nitric oxide (NO) synthase (NOS) inhibitor L‐NAME attenuated the hypertensive effects of H_2_S. C,D) Wire myography. Endothelium denudation did not alter the constrictive (C) or dilatory (D) effects of H_2_S on isolated sheep mesenteric arteries. *n* = 5. E) Coadministration of Na_2_S did not alter the hypotensive effects of acetylcholine (ACh) or S‐nitroso‐glutathione (GSNO). *n* = 5. Na_2_S was continuously infused resulting in a stable elevation in blood pressure before bolus injection of ACh or GSNO. F,G) GSNO coadministration blocked the hypertensive effects of H_2_S. *n* = 5. GSNO was continuously infused resulting a stable depression in blood pressure prior to bolus injection of Na_2_S. H–J) The LTCC inhibitor nifedipine blocked the H_2_S‐induced hypertensive effects (H and I) and constriction of isolated mesenteric arteries (J). *n* = 5. K) Schematic diagram showing the interaction between the NO‐cGMP pathway and H_2_S. (C) and (J) were performed in the presence of 1 µm phenylephrine. (D) was performed under the same conditions as Figure 1E. p value represents the results of 2‐way ANOVA of relative MAP increase versus intact sheep, shown in Figure 1B right. sGC = soluble guanylate cyclase, cGMP = cyclic guanosine monophosphate, PDE = phosphodiesterase, PKG = protein kinase G, eNOS = endothelial NOS.

Previous studies have proposed interactions between H_2_S and the NO‐cGMP pathway.^[^ ^3^ ^]^ To test whether H_2_S increases blood pressure via inhibition of endothelial NO synthase (eNOS), Na_2_S was administered 10 min following nonselective NOS inhibitor (L‐NAME) administration. L‐NAME rapidly suppresses eNOS activity, causing a swift rise in blood pressure that stabilizes within 5–10 min following administration (Figure S6, Supporting Information). The in vivo hypertensive effects of H_2_S were decreased (*p* = 0.0057; **Figure** **2**A,B) by L‐NAME, suggesting that the hypertensive effects of H_2_S involve NOS or its downstream effectors. However, neither the constrictive nor dilatory effects of H_2_S in isolated mesenteric arteries were altered when the endothelium underwent denudation (Figure 2C,D), ruling out the involvement of eNOS in the vasoactive effects of H_2_S. To test whether H_2_S potentiates or attenuates the NO‐cGMP pathway, the hypotensive effects of the endothelium‐dependent and endothelium‐independent NO‐cGMP pathway activators ACh and GSNO, respectively, were tested in vivo with and without Na_2_S coadministration. Na_2_S did not alter the dilatory effects of either drug, (Figure 2E), ruling out any significant effects of H_2_S, at the administered dose, on the NO signaling cascades, including the cGMP‐degrading phosphodiesterase (PDE). On the other hand, the hypertensive effects of H_2_S were not observed (Figure 2F,G) with the coadministration of GSNO, indicating that the signaling mechanism of the former involves a component that is blocked by the latter. GSNO has been shown to cause vasodilation via a pathway that inhibits the LTCC.^[^ ^22^ ^]^ We therefore tested the possibility that H_2_S‐mediated vasoconstriction involves activation of the LTCC. Consistent with this idea, the LTCC‐specific inhibitor nifedipine blocked the vasoconstricting effects of H_2_S in vivo and in vitro (Figure 2H–J). The proposed mechanisms for the interactions between the NO‐cGMP pathway and H_2_S, based on the results above, are summarized in Figure 2K. However, it is important to note that the current experiments only tested the interactions between Na_2_S and ACh/GSNO at specific doses. The full spectrum of the interactions remains to be explored, especially at physiological concentrations.

### 2.4 Reaction of H_2_S and Oxyhemoglobin Leads to a Hypertensive Intermediate

**Figure 3:**
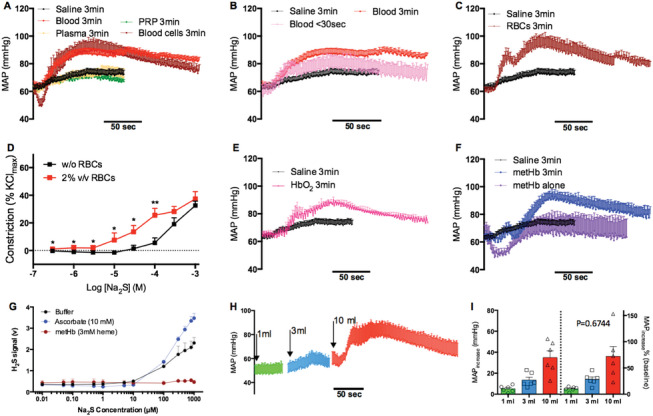
Reaction of H_2_S and blood leads to a hypertensive intermediate of H_2_S. A) The hypertensive effects of Na_2_S were potentiated by prior incubation with blood or blood cells but not plasma or platelet‐rich plasma (PRP). Three milliliters of each blood component was incubated with 3 mL of 128 mm Na_2_S for 3 min before injection. *n* = 5. B) Blood potentiated the hypertensive effects of Na_2_S in a time‐dependent manner. *n* = 5. C) Red blood cells (RBCs) potentiated the hypertensive effects of Na_2_S. *n* = 5. D) RBCs left‐shifted the constrictive dose response curve of Na_2_S in mesenteric arteries. Performed in the presence of 1 µm phenylephrine. *n* = 5. Paired t‐test. E) HbO_2_ (10 mm heme) potentiated the hypertensive effects of Na_2_S. *n* = 5. F) MetHb (3 mm heme) potentiated the hypertensive effects of Na_2_S. *n* = 5. G) Headspace detection of H_2_S as in Figure 1I. Parallel experiments showed that metHb effectively scavenged H_2_S, while ascorbate increased the H_2_S signal. *n* = 3. H,I) Methemoglobinemia did not alter the hypertensive effects of Na_2_S in vivo. All Na_2_S incubations were performed in a beaker open to air. *p* value represents the results of 2‐way ANOVA of relative MAP increase versus intact sheep, shown in Figure 1B right.

The hypertensive effects of Na_2_S persisted after H_2_S had disappeared from circulating blood (Figure 1G–I), suggesting that hypertension was not caused by H_2_S but rather by its metabolite or other byproduct(s). To test for the role of blood metabolism in the vasoactivity of H_2_S, Na_2_S was incubated with blood for 3 min prior to its intravenous administration. This prior incubation significantly potentiated the hypertensive effects of Na_2_S, suggesting that a hypertensive intermediate was generated from the reaction of H_2_S with some components of blood (**Figure** **3**A). A prior incubation of 30 sec was adequate to potentiate the hypertensive effects of Na_2_S to some extent, suggesting that H_2_S and blood rapidly reacted to generate the hypertensive intermediate (Figure 3B). The potentiation effects of blood were reproduced when H_2_S was incubated with washed blood cells containing platelets but not with isolated plasma, suggesting that the reactant involves the blood cells (Figure 3A). H_2_S has been suggested to activate intravascular release of serotonin,^[^ ^23^ ^]^ which platelets store at high concentrations and release upon activation.^[^ ^24^ ^]^ To test whether H_2_S raises blood pressure by activating the release of serotonin from platelets, Na_2_S was incubated with platelet‐rich plasma (PRP) prior to administration. Similar to plasma, PRP also failed to potentiate the hypertensive effects of H_2_S, ruling out the possibility that serotonin is released from platelets by H_2_S (Figure 3A).

Based on the above results, RBCs is the most likely site of H_2_S bioactivation in blood. Indeed, potentiation effects were observed after an incubation was performed with washed RBCs (Figure 3C). Consistent with these in vivo observations, RBCs also left‐shifted the constrictive dose response curve of Na_2_S in isolated arteries (Figure 3D). Interestingly, Na_2_S was found to hemolyze blood, resulting in cell‐free hemoglobin in a dose‐ and time‐dependent manner (Figure S7a,b, Supporting Information), which may scavenge NO and thus increase blood pressure. However, we have previously demonstrated that intravenous infusion of hemolysate does not alter MAP or systemic arterial resistance in sheep,^[^ ^25^ ^]^ suggesting that the hypertensive effects of H_2_S are not due to the hemolysate.

We next explored the role of RBC contents as a reactant in the production of the hypertensive intermediate of H_2_S. Prior incubation with 10 mM HbO_2_ potentiated the hypertensive effects of Na_2_S, suggesting that HbO_2_, which is the most abundant protein in RBCs, is one reactant (Figure 3E). Given that metHb generates various reactive sulfur species by reacting with H_2_S,^[^ ^16^, ^18^ ^]^ we also tested the effects of metHb on the hypertensive effects of H_2_S. Consistent with previous reports, metHb rapidly scavenges H_2_S (metHb:H_2_S = 1:3 ^[^ ^17^ ^]^), as measured by an H_2_S‐specific amperometric electrode (Figure 3G). Similar to HbO_2_, potentiation effects were also observed after incubation with 3 mm metHb, although the increase in MAP was somewhat delayed, probably by the transient hypotensive effects of metHb (Figure 3F). However, in contrast to the potentiation effects observed with prior incubation, in vivo methemoglobinemia caused by intravenous infusion of nitrite, which resulted in an increase in circulating metHb concentrations from 52±4 to 560±47 µm (Figure S8, Supporting Information), failed to potentiate the hypertensive effects of Na_2_S (Figure 3H,I). These results suggest that endogenous metHb played a limited role^–^ in the presence of 10 mm endogenous HbO_2_ as the reactant to produce the hypertensive intermediate of H_2_S.

### 2.5 Exclusion of HSOH, metHb‐SH^−^ Complexes, Persulfide, Polysulfide, and Thiolsulfate as the Hypertensive Intermediate

**Figure 4:**
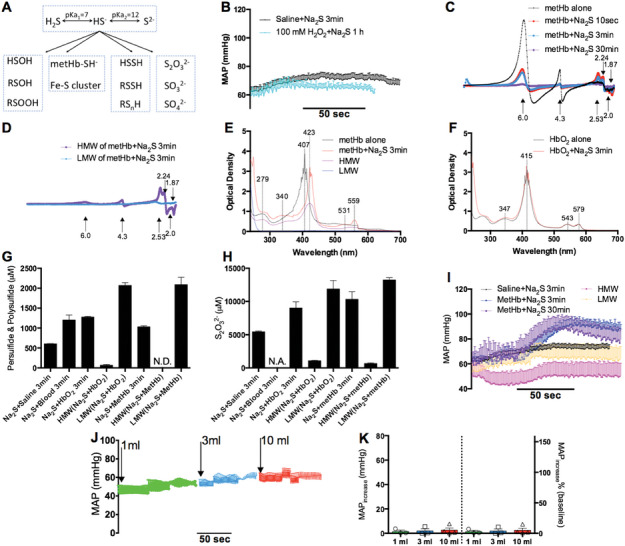
Exclusion of HSOH, metHb‐SH^−^ complexes, per‐/poly‐ sulfide, and thiosulfate as the hypertensive intermediate of H_2_S. A) Diagram for metabolism of H_2_S in blood. Protons are omitted for clarity. B) The hypertensive effects of Na_2_S were not potentiated by prior incubation with H_2_O_2_, which generates HSOH. *n* = 3. C,D) EPR detection of metHb‐SH^−^ complexes in the mixture of Na_2_S and metHb (C) and (D) its high molecular weight (HMW) and low molecular weight (LMW) fractions prepared by 25‐min G‐25 column separation. All spectra were normalized to the same scale. *n* = 3. E,F) UV‒VIS absorption spectra. (E) Reaction of Na_2_S and metHb generated metHb‐SH^−^ complexes with an absorption peak at 423 nm, whereas that of Na_2_S and HbO_2_ did not (F). *n* = 3. G,H) Cyanolysis assays of per‐/poly‐ sulfide and thiolsulfate. The reaction of Na_2_S and blood, HbO_2_, or metHb generated per‐/poly‐ sulfide (G) and thiolsulfate (H), which mostly remained within the LMW fraction. *n* = 3. I) Neither the HMW nor LMW fraction of the reaction mixture of Na_2_S and metHb was as hypertensive as the mixture. *n* = 5. J,K) Unlike Na_2_S, thiosulfate (intravenous injection of 3 mL bolus of 128 mm) administration did not increase MAP. *n* = 4. All Na_2_S incubations were performed in a beaker open to air.

The metabolism of H_2_S has been proposed to generate various products (**Figure** **4**A). For example, H_2_S reacts slowly with H_2_O_2_, a byproduct of HbO_2_ autoxidation,^[^ ^26^ ^]^ to generate hydrogen thioperoxide (HSOH).^[^ ^27^ ^]^ However, preincubation of Na_2_S with H_2_O_2_ for 1 h did not potentiate the hypertensive effects (Figure 4B), suggesting that HSOH is unlikely the hypertensive intermediate of H_2_S. MetHb has been shown to react with H_2_S to produce metHb‐SH^−^ complexes, persulfide, polysulfide, and thiosulfate.^[^ ^17^ ^]^ To test for the roles of these H_2_S metabolites as the hypertensive intermediate of H_2_S (Figure 3F), the HMW and LMW fractions of the reaction mixture of Na_2_S and metHb were isolated through G‐25 column passage, a process that took 25–30 min. Consistent with the formation of metHb‐SH^−^ complexes, an EPR signal (peaks with g factors at 2.53, 2.24, and 1.87; Figure 4C,D) and a UV‒VIS absorption spectrum (peak at 423 nm; Figure 4E) with the characteristics of metHb‐SH^−^ complexes ^[^ ^16^ ^]^ were observed in the reaction mixture and its HMW but not LMW fraction. However, the absorption peak of metHb‐SH^−^ complexes was not detected in the reaction mixture of Na_2_S and HbO_2_ or RBCs (Figure 4F; Figure S9, Supporting Information), suggesting little formation of the complexes in HbO_2_ or RBCs by H_2_S. These results excluded the metHb‐SH^−^ complexes as the hypertensive intermediate in the reaction mixture of Na_2_S and HbO_2_ or RBCs.

Consistent with previous reports,^[^ ^16^, ^17^ ^]^ poly‐/per‐sulfide and thiosulfate were detected by cyanolysis assays, in which reaction mixtures of Na_2_S and whole blood, oxyhemoglobin, or methemoglobin were incubated for three minutes, and in their LMW but not HMW fractions (Figure 4G,H). If one of these products was the hypertensive intermediate of H_2_S, the LMW fraction should have exhibited more potent hypertensive effects than that of Na_2_S. However, the potentiation effects were observed only with the whole reaction mixture containing Na_2_S and metHb, which was maintained for 30 min to control the time for G‐25 column fractionation, but not with either the LMW or HMW fraction (Figure 4I). Thiosulfate has been suggested to be a bioactive metabolite of H_2_S under some conditions.^[^ ^28^ ^]^ However, at the same dose of Na_2_S, thiosulfate did not demonstrate any hypertensive effects (Figure 4J,K). In sum, these results excluded all the above metabolism products as the hypertensive intermediate of H_2_S.

### 2.6 HS^•^ as the Hypertensive Intermediate of H_2_S, and the Role of Oxyhemoglobin

The reactions of H_2_S with HbO_2_^[^ ^29^ ^]^ and metHb ^[^ ^16^ ^]^ have also been suggested to involve the formation of HS**^•^**. We therefore measured this radical with EPR using BMPO, DMSO, and SOD1 as a spin trap, hydroxyl scavenger, and superoxide scavenger, respectively, as previously described.^[^ ^30^ ^]^ Further validation of this methodology is provided in Figure S10 (Supporting Information).

**Figure 5:**
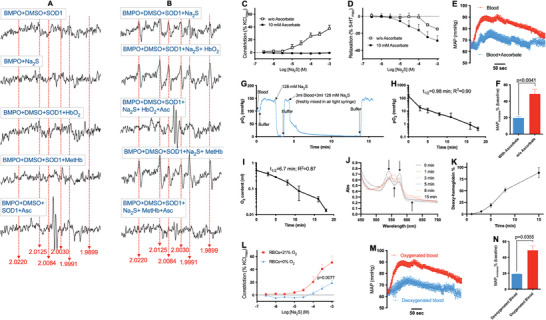
HS^•^ is the hypertensive intermediate in the reaction of H_2_S and oxyhemoglobin (HbO_2_). A,B) Representative EPR spectra. *n* = 3. Reaction of H_2_S and HbO_2_/metHb generated HS^•^ that can be scavenged by ascorbate. HS^•^ was measured with EPR via the use of BMPO as a spin trap, which results in characteristic peaks at the g‐factors indicated by the vertical red lines. DMSO was used as a hydroxyl scavenger, and SOD1 was used as a superoxide scavenger. All traces are shown under the same y‐axis scale and aligned by g‐factor. Note that samples with Na_2_S in the presence of HbO_2_ or methHb resulted in the most abundant HS^•^ production. (C‐D) In isolated mesenteric arteries, ascorbate blocked C) and potentiated D) H_2_S‐induced constriction (C; same condition as Figure 3D with the presence of 2% v/v RBCs) and relaxation (D; same condition as Figure 1E), respectively. *n* = 5. E,F) Coincubation with 150 mM ascorbate (mixed with 128 mM Na_2_S and blood for 3 min; 3 mL each) attenuated the potentiation effects of blood on Na_2_S‐induced hypertensive effects. *n* = 5. G–K) H_2_S consumes O_2_ in blood. *n* = 4. Three milliliters of 128 mM Na_2_S and 3 ml blood (HbO_2_ for J‐K) were incubated in an airtight syringe. (G) Na_2_S decreased blood pO_2_. A fluorometric O_2_ probe was successively inserted into different solutions for measurements of pO_2_. HEPES buffer was exposed to air as the positive control. (H‐I) Time‐dependent decreases in blood pO_2_ (H) and oxygen saturation (I) by Na_2_S as measured by a blood gas analyzer. R^2^ is for fit of the data to a one phase exponential decay equation. (J‐K) UV‒VIS measurement of deoxyhemoglobin formation in HbO_2_ during incubation with Na_2_S. (J) UV‒VIS absorption spectra. K) Fraction of deoxyhemoglobin. L) Influence of blood‐borne O_2_ on H_2_S‐mediated vessel constriction in the presence of 1 µm phenylephrine and RBCs sparged with 21% or 0% O_2_ in a balance of N_2_. n = 5. Two‐way ANOVA. M,N) Influence of blood‐borne O_2_ on Na_2_S‐induced hypertensive effects. *n* = 5. M) Averaged MAP traces. N) Relative increase in MAP. Pre‐incubation with oxygenated blood markedly potentiates the Na_2_S‐induced hypertensive effects compared to deoxygenated blood (incubated in an airtight syringe). Paired *t*‐test.

As shown in **Figure** **5**A,B, an EPR signal characteristic of BMPO‐trapped HS**^•^** ^[^ ^29^, ^31^, ^32^ ^]^ was generated from the addition of Na_2_S. Consistent with their ability to catalyze the production of HS**^•^** as the hypertensive intermediate of H_2_S, HbO_2_ and metHb augmented the EPR signal of HS**^•^** (Figure 5B). This effect was eliminated when free radical scavenger sodium ascorbate (Figure 5A,B) was added, which does not scavenge H_2_S (^[^ ^33^ ^]^ and Figure 3G). These results provide evidence for the presence of HS**^•^** as an intermediate in the chemical reaction of HbO_2_ or metHb with H_2_S and the successful use of ascorbate as a scavenger of HS**^•^**. To further test the role of HS**^•^** as the hypertensive intermediate of H_2_S, the functional effects of HS**^•^** scavenging by ascorbate were tested. Ascorbate completely blocked the Na_2_S‐induced contraction of isolated arteries in the presence of RBCs (Figure 5C). In addition, ascorbate left‐shifted the dilatory dose response curve of Na_2_S (Figure 5D), possibly by unmasking the dilatory effects of H_2_S through eliminating concomitant vasoconstriction by HS**^•^**. Consistent with HS**^•^** scavenging, ascorbate, which did not alter blood pressure when applied alone (Figure S11, Supporting Information), eliminated the potentiation effects of prior incubation of blood on the hypertensive effects of Na_2_S in vivo (Figure 5E,F). These results strongly support the role of HS**^•^** as the hypertensive intermediate of H_2_S.

The one‐electron oxidation of H_2_S to HS**^•^** in blood requires an electron acceptor, i.e., oxidant. The reaction of H_2_S with metHb has been shown to consume oxygen.^[^ ^17^ ^]^ Na_2_S at millimolar levels decreased the partial pressure of oxygen (pO_2_) in HEPES buffer, as measured by a fluorometric O_2_ probe (Figure S12, Supporting Information). We therefore used this probe to test whether the production of HS**^•^** from H_2_S in whole blood or HbO_2_ solution consumes O_2_. Consistent with this idea, under airtight conditions, a decrease in blood pO_2_ was detected after Na_2_S was added (Figure 5G). The decrease was also observed in the presence of ascorbate, ruling out the consumption of O_2_ by HS**^•^** (Figure S13, Supporting Information). In addition, blood pO_2_ and oxyhemoglobin saturation were decreased by Na_2_S, as measured by a blood gas analyzer that performs amperometric measurement of pO_2_ and spectrophotometric measurement of HbO_2_ (Figure S14a,b, Supporting Information). Both pO_2_ and total O_2_ content decreased, with the presence of an abundance of Na_2_S over O_2_ (128 vs ≈10 mm), with the kinetic characteristics of a first‐order reaction (Figure 5H,I). In addition, the deoxygenation of HbO_2_ by reaction with Na_2_S was confirmed with UV‒VIS spectrophotometry (Figure 5J,K). These results all support the role of blood‐borne O_2_ as the oxidant in the one‐electron oxidation of H_2_S.

We next conducted wire myography experiments to examine the influence of O_2_ on H_2_S‐mediated vessel constriction in the presence of RBCs while sparging the vessel baths with either room air or 100% N_2_ (Figure 5L). These parallel experiments demonstrated that 21% O_2_ markedly potentiated the H_2_S‐mediated vessel constriction compared to N_2_, underscoring the pivotal role of O_2_ in facilitating H_2_S oxidation and vasoconstriction. In addition, we conducted a comparative analysis of the hypertensive effects of Na_2_S when pre‐mixed with deoxygenated and oxygenated blood prior to intravenous injection. As illustrated in Figure 5M,N, Na_2_S pre‐mixed with deoxygenated blood resulted in a modest 20% increase in mean arterial blood pressure, which did not exhibit greater potency in inducing hypertension compared to Na_2_S dissolved in buffer alone (Figures 1B and 3A). These results suggest that deoxygenated blood alone does not contribute to the hypertensive effects of H_2_S, indicating a lack of participation in the H_2_S oxidation process. Furthermore, when Na_2_S was pre‐mixed with oxygenated blood, a substantial 45% increase in mean arterial blood pressure was observed, demonstrating a significantly more potent hypertensive response compared to pre‐mixing Na_2_S with deoxygenated blood or buffer. These results align with our in vitro observations and emphasize the indispensable role of blood‐borne O_2_ in facilitating H_2_S oxidation and its consequent hypertensive effects.

### 2.7 Activation of LTCC by HS^•^

**Figure 6:**
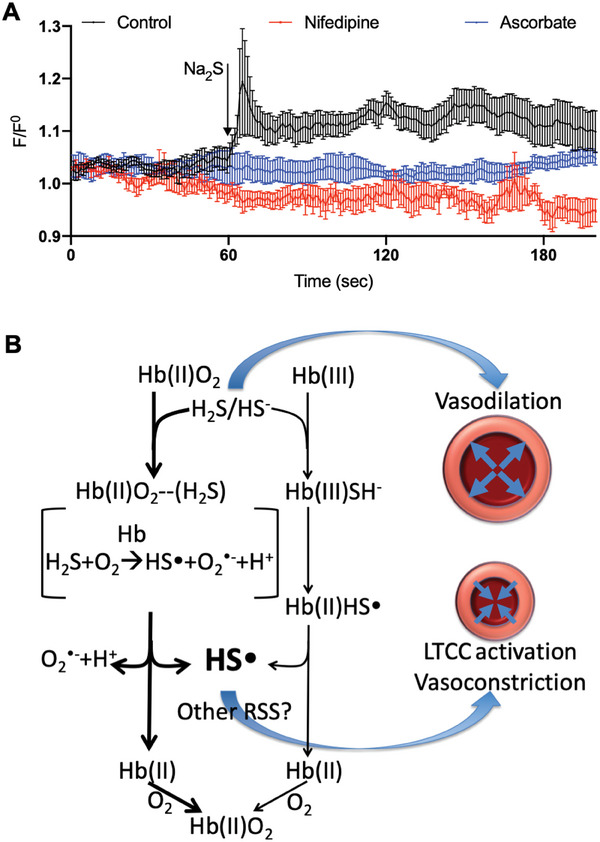
Mechanisms underlying the hypertensive effects of Na_2_S. A) The LTCC blocker nifedipine and HS^•^ scavenger ascorbate both blocked the Na_2_S‐induced increases in Ca^2+^ signal measured by confocal microscopy. *n* = 5. Experiments were performed in the presence of 1 µm phenylephrine. B) Diagram for the mechanisms underlying the hypertensive effects of H_2_S.

We further explored the mechanisms underlying the hypertensive effects of HS**^•^**. Consistent with the above functional observations that the LTCC blocker nifedipine blocked the vasoconstricting effects of Na_2_S (Figure 2H–J), nifedipine also blocked the Na_2_S‐induced increases in Ca^2+^ signal, as measured fluorometrically via confocal microscopy; this result further confirmed that activation of LTCC plays a role in the hypertensive effects of Na_2_S. Similar to nifedipine, the HS**^•^** scavenger ascorbate blocked the Na_2_S‐induced increases in Ca^2+^ signal (**Figure** **6**A; Figure S15, Supporting Information), implicating HS**^•^** in the activation of the LTCC. In addition, Na_2_S‐induced vasoconstriction was inhibited in the presence of the disulfide bond breakers TCEP and DTT and by prior exposure of the isolated arteries to the thiol oxidizers diamide and MMTS (Figure S16, Supporting Information), indicating that redox‐sensitive thiol(s) are involved in the activation of LTCC by HS**^•^**. The possible pathways for the production of hypertensive HS**^•^** from H_2_S in blood and the mechanism underlying the hypertensive effects of H_2_S are shown in Figure 6B.

## 3 Discussion

### 3.1 What is the Hypertensive Agent?

While H_2_S was previously found to exhibit vasodilatory and vasoconstricting effects, our results demonstrate an overriding vasoconstricting response in our experimental models. Evidence suggesting that the vasoconstriction effects were not mediated directly by H_2_S. First, the hypertension that followed intravenous injection of Na_2_S persisted after H_2_S was eliminated from the circulating blood (Figure 1). Second, free H_2_S is rapidly scavenged by metHb,^[^ ^16^, ^17^, ^18^ ^]^ and thus, methemoglobinemia should attenuate the hypertensive response to Na_2_S infusion if H_2_S causes vasoconstriction. However, methemoglobinemia showed no significant effect on the hypertensive effects of Na_2_S infusion even though the circulating methemoglobin could adequately scavenge all administered H_2_S. Thus, it is likely that the hypertensive effects that followed Na_2_S infusion were due to a metabolite of H_2_S rather than H_2_S. The predominant metabolites of H_2_S include HSOH, metHb‐SH^−^ complexes, persulfide, polysulfide, and thiolsulfate. Nevertheless, as described above, their roles as the hypertensive mediator of H_2_S were also excluded by the current experiments.

Hypertensive metabolite requires the simultaneous presence of the HMW and LMW fractions, suggesting the possibility that the hypertensive metabolite is a labile product of a reaction between HMW and LMW substrates. The product of HS^−^ oxidation, thiyl radical (HS**^•^**),^[^ ^32^ ^]^ is one possible candidate that fits this criteria. Using EPR, we found measurable levels of HS**^•^** that were produced from the incubation of Na_2_S with HbO_2_ or metHb (Figure 5B). This is consistent with the observations that preincubation of Na_2_S with RBCs, HbO_2_, or metHb potentiates the hypertensive effects of Na_2_S infusion (Figure 3C,F) and that RBCs potentiate the vasoconstrictive effects of Na_2_S on isolated arteries (Figure 3D,G). Sodium ascorbate, which eliminates HS**^•^** (Figure 5B) by converting it to H_2_S and does not scavenge H_2_S (Figure 3G and ^[^ ^33^ ^]^), abolished the Na_2_S‐induced contraction of isolated arteries in the presence of RBCs (Figure 5C), unmasked the vasodilatory effects of Na_2_S on preconstricted arteries (Figure 5D), and blocked the potentiating effects of prior blood incubation on Na_2_S‐induced hypertension (Figure 5E,F). These results suggest the essential role of HS**^•^** in the vasoconstricting effects of H_2_S.

Notably, to our knowledge, no HS**^•^**‐specific scavenger is available, and we acknowledge the possibility of confounding off‐target effects of sodium ascorbate, such as partial reduction of metHb into Hb and scavenging of other radicals. The labile HS**^•^** likely leads to a variety of downstream reactive sulfur species (RSS),^[^ ^34^, ^35^ ^]^ such as per‐/poly‐sulfide radicals. The vasoactivity of these RSSs and their role in the vasoconstricting effects of H_2_S deserve further study.

It is important to highlight that pH perturbations are not a significant concern in the current study. While Na_2_S has alkalinity, reactions of H_2_S may result in the generation of H^+^ (Figure 6B), suggesting a potential acidification. In our sheep model, the circulatory Na_2_S concentration is calculated to be only 350 µm at the highest dose (10 mL of 128 mm) of Na_2_S, even if diffusion into tissue and metabolism are not taken into account. Na_2_S as high as 1 mm does not significantly alter the pH of HEPES buffer (10 mm; pH = 7.40) or sheep blood (Figure S17a,b, Supporting Information) in vitro. Consistent with this, blood gas measurements (Figure S17c, Supporting Information) demonstrated that Na_2_S administrations did not change blood pH in vivo. In addition, Na_2_S and NaHS, a neutral donor of H_2_S, have similar H_2_S releasing properties in HEPES buffer (Figure S18, Supporting Information). Furthermore, consistent with previous reports that both Na_2_S and NaHS generate HS**^•^**,^[^ ^29^, ^32^ ^]^ our EPR measurements demonstrated that, like Na_2_S, NaHS also generates HS**^•^** by reaction with HbO_2_ and metHb (Figure S19, Supporting Information).

### 3.2 How does HS^•^ Mediate Hypertensive Effects?

The current study suggests that HS**^•^**‐mediated vasoconstriction involves activation of the LTCC. Reports on the mechanisms that mediate the hypertensive effects of H_2_S have been contradictory. For example, although H_2_S has often been reported as an inhibitor of the LTCC, it can also activate the LTCC.^[^ ^36^, ^37^ ^]^ Similarly, H_2_S has been demonstrated to inhibit endothelial NO synthase ^[^ ^6^, ^38^ ^]^ and stimulate the release of endothelial NO.^[^ ^6^, ^38^ ^]^ As will be discussed, these discrepant results may be explained by considering whether the effector was H_2_S or its redox cousin HS**^•^**. Similar to what was previously demonstrated for other redox cousins (HNO and O_2_ **^•^** ^−^) of NO^[^ ^39^, ^40^ ^]^ and O_2_,^[^ ^39^, ^40^ ^]^ HS**^•^** and H_2_S may exhibit distinct biochemical properties.^[^ ^34^ ^]^ While the current results indicate that the LTCC is essential for the vasoconstricting effects of HS**^•^**, we can only speculate on the specific mechanism. It is possible that HS**^•^** generated from H_2_S may posttranslationally modify one or more redox‐sensitive thiols on proteins involved in the contractile pathway leading to or including the LTCC, causing the channel to open. Consistent with the involvement of redox‐sensitive thiols, Na_2_S‐induced constriction was inhibited in the presence of disulfide bond breakers and by the prior exposure of the isolated arteries to thiol oxidizers (Figure S16, Supporting Information). Further investigation into the mechanism of HS**^•^**‐mediated LTCC activation is necessary.

### 3.3 How is H_2_S Metabolized in Blood?

In this study, we demonstrated that 128 mm Na_2_S eliminated >10 mm O_2_ from blood when mixed at a volume ratio of 1:1 (Figure 5G–K), suggesting the oxidation of H_2_S by O_2_ in blood. Although the direct reaction of H_2_S with O_2_ is likely too slow to account for these results,^[^ ^14^, ^15^ ^]^ some metalloproteins, such as metHb, are capable of catalyzing H_2_S oxidation.^[^ ^16^, ^17^, ^18^, ^41^ ^]^ However, based on a reported stoichiometry that 1 mole of metHb can oxidize 3 moles of H_2_S,^[^ ^17^, ^41^ ^]^ metHb, which is measured at ≈52 µm in sheep blood, is only capable of oxidizing up to 156 µm H_2_S. Thus, if the oxidation of H_2_S by metHb consumes one O_2_ per H_2_S, the 156 µm of O_2_ consumed would be inadequate to explain the observed consumption of >10 mm of O_2_. This gap suggests the presence of a previously undescribed pathway for H_2_S oxidation. Given that deoxyHb was generated from HbO_2_ during the process of H_2_S oxidation (Figure 5J–K), the reaction may occur at the site of oxygenated heme, i.e., HbO_2_ directly participates in H_2_S oxidation. This possibility is supported by the observation of HS**^•^** generation facilitated by the presence of HbO_2_ (Figure 5B) and the accompanying facilitation of H_2_S‐mediated vasoconstriction by oxygenated RBCs in organ baths (Figure 3D). In addition, in contrast to the potentiation of the hypertensive effects of Na_2_S by prior incubation with metHb (Figure 3F), methemoglobinemia, in which circulatory metHb increased from 52 to 560 µm, failed to potentiate the hypertensive effects of Na_2_S infusion (Figure 3H,I). These results indicated that the circulatory reactant for the generation of HS**^•^** was present at concentrations that far exceeded 560 µm metHb, also agreeing with the involvement of 10 mm circulatory HbO_2_ in H_2_S oxidation. Therefore, this study suggests a novel role for hemoglobin, i.e., the oxidation of H_2_S, which occurs when hemoglobin is in a ferrous and O_2_‐bound form. Based on the first‐order reaction kinetics of O_2_ consumption and reported indications,^[^ ^16^, ^17^, ^18^, ^32^ ^]^ the reaction equation is proposed as **H_2_S + O_2_ → HS^•^ + O_2_^•−^** + H^+^ (Figure 6B), with ferrous heme Hb as the catalyst, although further studies are warranted to validate the production of O**_2_** ^•−^ and H^+^.

### 3.4 Hypertensive Versus Hypotensive: What Determines the Vasoactive Effect of H_2_S?

As introduced above, many factors, including O_2_ concentration, have been demonstrated to affect the vasoactivity of H_2_S.^[^ ^4^ ^]^ Two possible mechanisms have been proposed to explain the role of O_2_ in determining the vasoactive responses to H_2_S.^[^ ^42^, ^43^ ^]^ Based on the observations that the same concentrations of H_2_S mediate contraction at high O_2_ levels but relaxation at lower O_2_ levels in rat aorta, Kraus et al. proposed that vasodilatory H_2_S generates a postulated vasoconstrictive oxidation product at high O_2_ that competes with H_2_S‐mediated relaxation.^[^ ^43^ ^]^ However, in some vessels, H_2_S is a monophasic vasoconstrictor and its constriction is facilitated by *lower* levels of O_2_.^[^ ^42^ ^]^ Based on the prevalent findings that H_2_S and hypoxia have temporally and quantitatively identical vascular responses and that the tissue concentration of H_2_S is inversely coupled to O_2_ levels owing to its O_2_‐dependent metabolism, Olson et al. proposed the theory that H_2_S serves as an oxygen sensor and transducer of vascular responses to hypoxia. This theory stipulates that the varying vasoactive effects of H_2_S are an intrinsic property of vascular smooth muscle cells.^[^ ^19^, ^42^, ^44^ ^]^

The current study proposes that HS**^•^**, the one electron oxidation product of H_2_S, is a hypertensive intermediate of H_2_S that competes with the vasodilatory effects of H_2_S (Figure 6B). At first glance, our proposal and Olson's differs; however, we supported the proposal of a vasoconstrictive oxidation product ^[^ ^43^ ^]^ by demonstrating that the oxidation product of H_2_S is indeed vasoconstrictive and O_2_‐dependent.^[^ ^43^ ^]^ Nevertheless, the two proposals might be reconciled because tissue‐specific vasoactive responses to H_2_S may be due to varying intrinsic abilities of the vascular smooth muscle to oxidize H_2_S. Consistent with the possibility of intrinsic properties of the vessel playing a role, we observed that exposure of isolated arteries to basal levels of phenylephrine, which may increase H_2_S oxidation in vessels in a dose‐dependent manner,^[^ ^43^, ^45^ ^]^ dose‐dependently facilitated constriction by H_2_S in the isolated arteries (Figure 1F). Further studies are needed to test uncertainties, such as whether pulmonary and systemic arteries utilize distinct routes for O_2_ handling/ROS production ^[^ ^46^, ^47^ ^]^ and thus different RSSs ^[^ ^19^ ^]^ and whether the inhibition of H_2_S oxidation results in the widely reported transition of H_2_S‐induced vasoconstriction to vasodilation at higher H_2_S levels.^[^ ^5^, ^6^, ^7^, ^8^ ^]^

### 3.5 Is This Study Physiologically Relevant?

In the current study, H_2_S caused 10% contraction at 10 µm in isolated arteries and increased the MAP by 11% at a calculated circulatory concentration of 35 µm at the 1 mL bolus, although it was rapidly transformed into undetectable metabolites in blood. Despite the establishment of H_2_S as a gasotransmitter, the physiological concentration of circulatory H_2_S has not yet been established.^[^ ^48^, ^49^ ^]^ Early reports of circulatory H_2_S lie in the micromolar range, which is also the level of H_2_S needed for vasoactivity in isolated arteries. Nevertheless, recent studies have indicated that circulatory H_2_S is in the low nanomolar range,^[^ ^50^ ^]^ casting doubt on the vasoactive signaling function of physiological H_2_S. It has been suggested that concentrations of H_2_S below 100 µm in wire myography could be within the physiological range ^[^ ^50^, ^51^ ^]^ and that the mid‐micromolar range of the circulatory pool of H_2_S metabolites rather than H_2_S may be pertinent to H_2_S‐based signaling.^[^ ^27^, ^48^, ^52^, ^53^ ^]^ From these points of view, the current observations are physiologically relevant. Intriguingly, the scavenging of HS**^•^** by sodium ascorbate left‐shifted the dilatory dose response curve of Na_2_S, possibly by unmasking the dilatory effects of H_2_S, suggesting that HS**^•^** may be more potent than H_2_S in the regulation of vascular tone.

## 4 Conclusions

In sum, this study proposes that single electron oxidation of H_2_S by oxyhemoglobin generates an HS**^•^**, which causes vasoconstriction via an LTCC‐dependent pathway and thus increases systemic arterial blood pressure. The findings of the current study have implications for the role of H_2_S in vascular physiology, pathology, and toxicology.

## 5 Experimental Section

### Materials

S‐nitroso‐glutathione (GSNO) was synthesized as previously described.^[^ ^22^ ^]^ Details are provided in the supplemental materials. ^34^S‐labeled Na_2_S was synthesized from ^34^S powder (Cambridge Isotope Laboratories, Andover, MA) as previously described.^[^ ^12^ ^]^ Briefly, ^34^S powder and zinc dust were heated to 170 °C for 1.5 h in an argon‐purged reaction vessel to generate Zn^34^S. Then, H_2_^34^S was produced by reacting ground Zn^34^S with HCl, and ^34^S was captured as Na_2_^34^S by passing through NaOH solution. 5‐*tert*‐Butoxycarbonyl‐5‐methyl‐1‐pyrroline‐N‐oxide (BMPO) was purchased from Enzo Life Sciences (Farmingdale, NY). β‐(4‐hydroxyphenyl)ethyl iodoacetamide (HPE‐IAM) was obtained from Avantor (Edmonton, Alberta, Canada). Fluo‐4 AM was purchased from Invitrogen (Carlsbad, CA). HbO_2_ was prepared by passing the lysed and washed arterial red blood cells (RBCs) through a Sephadex G‐25 column with an exclusion limit of 5 kDa. Other chemicals, such as metHb powder, were obtained from Sigma Aldrich (St Louis, MO). Na_2_S (128 mm unless specifically stated) was freshly prepared with HEPES buffer (pH = 7.4; containing 0.1 mm diethylene triamine pentaacetic acid (DTPA)). At pH 7.4, 18.6% of Na_2_S occurs as H_2_S in the equilibration of H_2_S/HS^−^/S^2−^, which is collectively called H_2_S below.

### Surgical Preparations and Infusion Protocols

All procedures (IACUC 21–196 & 21–198) involving animals were performed according to the National Institutes of Health Guide for the Care and Use of Laboratory Animals and were preapproved by the Loma Linda University (LLU) Institutional Animal Care and Use Committee.

### Sheep Protocol

Western breed pregnant ewes (55.3 ± 1.8 kg) between 1 and 3 years old were studied between 138 and 143 days of gestation (term 150 days, Nebeker Ranch; Lancaster, CA). Anesthesia was induced by intravenous administration of ketamine (10 mg kg^−1^) and midazolam (0.5 mg kg^−1^) followed by intubation and ventilation with 1.5 to 2.5% isoflurane in O_2_ for the duration of surgical instrumentation and until the end of the experiment. The fetuses were delivered via cesarean section and used in other unrelated experiments. The uterus was returned to the abdomen, and the abdominal incision sutured shut. In the ewe, catheters were inserted in both brachial arteries and in brachial veins to obtain blood samples, perform infusions, and measure arterial blood pressure. Chemicals were injected intravenously as boluses through separate catheters unless specifically stated.

### Na_2_S Infusion and Interventions

After a stable baseline period, the vasoactivity of H_2_S was studied by injecting (within 3 s) intravenous boluses of 1, 3, and 10 mL of 128 mm Na_2_S consecutively at 3‐minute intervals. To test for possible mechanisms underlying the vasoactive effects of Na_2_S boluses, ewes were assigned to one of three groups that received either a drug treatment, carotid chemoreceptor denervation, or no intervention (Control) prior to the infusing of Na_2_S. The drug treatments included the ganglionic activity blocker hexamethonium (1.6 mg kg^−1^), alpha‐1 adrenergic receptor antagonist prazosin (0.4 mg kg^−1^), COX1/2 inhibitor indomethacin (1 mg kg^−1^), NO synthase inhibitor L‐NAME (32 mg kg^−1^), cGMP pathway activator GSNO (0.8 mg kg^−1^ h^−1^), cAMP pathway agonist isoproterenol (40 µg kg^−1^ + 12 µg kg^−1^ h^−1^), K_ATP_ channel blocker glibenclamide (0.4 mg kg^−1^), LTCC inhibitor nifedipine (0.5 mg kg^−1^), and methemoglobinemia inducer sodium nitrite (4 mg kg^−1^). The carotid chemoreceptors were denervated surgically as previously described.^[^ ^54^ ^]^

### Na_2_S_2_O_3_ Infusion

The vasoactivity of thiosulfate (Na_2_S_2_O_3_), a predominant metabolite of H_2_S in blood, was tested in the same 1, 3, and 10 mL 128 mm rising‐dose bolus manner described above for Na_2_S.

### Acetylcholine and GSNO Infusion

To test the effects of H_2_S on the NO‐cGMP pathway, the vasoactivity of the endothelium‐dependent and endothelium‐independent cGMP activators acetylcholine (ACh; 1 mL of 0.7 mm) and GSNO (1 mL of 5 mm) were examined in the presence and absence of coadministration of Na_2_S (continuous infusion of 128 mm Na_2_S at 1 mL min^−1^ started 5 min prior to ACh or GSNO bolus).

### Incubation of Na_2_S with Blood Components Prior to Infusion

To test the role of H_2_S metabolism in blood in the vasoactivity of H_2_S, 3 mL of 128 mm Na_2_S was incubated at room temperature in a beaker (unless specifically stated) with 3 mL of various components of blood for 3 min (unless specifically stated) before injection or other experiments. The blood components included whole blood (arterial blood collected from intact ewes), plasma, blood cells (plasma replaced with saline), platelet‐rich plasma (PRP; prepared by centrifuging whole blood at 120 g for 15 min), red blood cells (RBCs; prepared by replacing PRP with saline), HbO_2_, and metHb (3 mm heme). For some injections, 3 mL of 150 mm sodium ascorbate was added for incubation. High molecular weight (HMW) and low molecular weight (LMW) fractions of some of the above reaction mixtures were separated after 3 min of incubation via G‐25 columns for injection or other experiments.

### Rat Protocol

Female nonpregnant Sprague‒Dawley rats weighing 301±5 g were surgically instrumented as previously reported ^[^ ^55^ ^]^ for testing the vasoactivity of H_2_S. Briefly, anesthesia was induced by 2.5% isoflurane in O_2_ and maintained with an intraperitoneal injection of urethane (800 mg kg^−1^) under spontaneous respiration of room air. Arterial blood pressure was measured through a catheter inserted into one carotid artery. A Na_2_S bolus (0.5 mL of 12.8 mm) was injected into the jugular vein over 3 s.

### Wire Myography

Sheep mesenteric and femoral arteries were dissected from ewes and mounted in organ bath chambers as previously described.^[^ ^56^ ^]^ To test for dilatory effects, vessels were first constricted with 10 µm serotonin (5‐HT) and then subjected to accumulating additions of Na_2_S. To test for contractile effects, vessels were first subjected to accumulating additions of Na_2_S, washout, and finally constriction with 125 mm KCl. In some experiments, 0.1 or 1 µm phenylephrine (PE), 2% (v/v) RBCs, 10 mm sodium ascorbate, 10 µm nifedipine, 100 µm diamide, 100 µm S‐methyl methanethiosulfonate (MMTS), 200 µm tris(2‐carboxyethyl)phosphine (TCEP), or 200 µm dithiothreitol (DTT) was added before tuning for basal tension (1.0 gram) and Na_2_S addition. Diamide and MMTS were washed out 15 min after they were added, while all other additives remained in the bath until the end of the accumulating additions of Na_2_S. The contractile effects of phenylephrine were tested in the same manner as Na_2_S. The endothelium was intact unless specifically stated otherwise.

### Ca^2+^ Imaging by Confocal Microscopy

Isolated sheep mesenteric arteries were denuded of endothelium, loaded with the Ca^2+^‐sensitive fluorescence dye Fluo‐4 AM (10 µm), and pinned to a Sylgard block for imaging with a Zeiss 710 laser scanning confocal microscope as previously described.^[^ ^57^ ^]^ Three arterial segments from one animal were imaged each day. The first segment (control group) was perfused successively with HEPES buffer containing 1 µm PE and then 1 µm PE with 100 µm Na_2_S with each perfusion lasting 5 min. The second (nifedipine group) and third (ascorbate group) segments were perfused in the same manner as the first segment but with the addition of 10 µm nifedipine and 10 mm ascorbate, respectively. The fluorescence signal was quantified using the LC‐pro plugin of ImageJ version 1.53k.

### Pharmacokinetic Analyses of H_2_S

The metabolism kinetics of H_2_S were measured in vivo and in vitro. For in vivo studies, sheep venous blood samples (1 mL each) were collected into heparinized syringes at 1, 2, 3, 4, 5, 8, and 12 min after the 10 mL injection of 128 mm Na_2_S. Plasma was separated from blood by centrifugation at 12 000 rpm for 30 s, snap frozen in liquid nitrogen, and stored at −80 °C until analysis. For in vitro studies, Na_2_S was injected to an initial concentration of 100 µm in heparinized venous sheep blood. Plasma was then isolated at 1, 2, 3, 4, 5, 8, 12, and 15 min and analyzed immediately.

### H_2_S Measurements by HPLC‒MS^2^

Plasma H_2_S concentrations were measured by HPLC‒MS^2^ in similar manner to a previous report.^[^ ^58^ ^]^ Details are provided in the supplemental materials. Briefly, 50 µl of plasma was mixed with 200 µL of methanol solution containing 5 mm HPE‐IAM (derivatization reagent) and 5 µm ^34^S‐labeled Na_2_S (internal standard) and derivatized at 37 °C for 30 min. The supernatant was collected for measurement (Figure S1, Supporting Information) after deproteinization via centrifugation. The standard curve samples (0.1–100 µm) of Na_2_S were generated with a deoxygenated HEPES buffer.

### H_2_S Measurements by H_2_S‐Specific Probe

Gaseous H_2_S was detected via a H_2_S‐specific amperometric electrode (ISO‐H2S‐2; WPI Inc.; Sarasota, FL) connected to a Free Radical Analyzer (TBR4100; WPI Inc.). Thirty milliliters of HEPES buffer or buffer containing metHb (3 mm heme) or sodium ascorbate (10 mm) was continuously stirred in a 50 mL Erlenmeyer flask sealed with parafilm. Accumulating concentrations of Na_2_S solution were injected into the bottom of the flask via a catheter. H_2_S that escaped out of the buffer was measured via a probe placed in the headspace.

### Measurements of Sulfane Sulfur

Sulfane sulfur was detected by cyanolysis assays as previously described.^[^ ^17^ ^]^ Persulfide and polysulfide were quantified by cold cyanolysis, while thiosulfate was quantified by subtraction of the measurements of cold cyanolysis from hot cyanolysis, which additionally detects thiosulfate.

### Electron Paramagnetic Resonance (EPR) Measurements

EPR signals were recorded using a Bruker X‐Band EMX Plus EPR spectrometer with a cavity of high sensitivity as previously described.^[^ ^59^ ^]^ The EPR was set to a microwave power of 20 mW, microwave frequency of 9.34 GHz, attenuator of 10 dB, modulation amplitude of 1 G, modulation frequency of 100 kHz, time constant of 20.48 msec, conversion time of 81.92 msec, harmonic of 1, and number of scans of 2. For measurements of HS**^•^**, BMPO (25 mm), Na_2_S (25 mm), DMSO (500 mm), SOD1 (1000 U mL^−1^), HbO_2_ (2 mm heme), metHb (600 µm), and/or sodium ascorbate (10 mm) were mixed in HEPES buffer and measured at 6.0 min from the initiation of the reaction. MetHb‐SH^−^ complexes were measured at 100 K, while HS**^•^** was measured at 293 K.

### Measurements of metHb, Hemolysis, Deoxyhemoglobin, Partial Pressure of Oxygen (pO_2_), and Oxyhemoglobin Saturation

MetHb and heme concentrations were determined using Drabkin's reagents ^[^ ^60^ ^]^ to evaluate methemoglobinemia and hemolysis, respectively. Video of H_2_S‐induced hemolysis was recorded under microscopy using a digital camera (Supplemental Vedio1). A UV‒visible spectroscopic scan was performed on a Varian Cary 50 (Agilent Technologies, Inc., Santa Clara, CA). The percentage of deoxyhemoglobin was calculated via deconvolution of the UV‒VIS spectrum by multiple linear regression analysis using basis spectra for oxyhemoglobin, deoxyhemoglobin, metHb, nitrosyl‐hemoglobin, and carboxyhemoglobin as described before ^[^ ^61^ ^]^ The partial pressure of oxygen (pO_2_) in some experiments was measured by a fluorometric O_2_ probe (OxyLite Pro XL; Oxford Optronix Ltd. Abingdon, UK). Blood pO_2_ and oxyhemoglobin saturation were measured by a blood gas analyzer (ABL 800, Radiometer, Copenhagen).

### Statistics

Average values are given as the mean ± SEM. Two‐way ANOVA was used to compare responses in the different infusion groups. Paired t tests were used as appropriate. Statistical analyses were carried out with Prism v8.4.0 (GraphPad Software, La Jolla, CA) with significance accepted at *p* < 0.05.

## Conflict of Interest

The authors declare no conflict of interest.

## Author Contributions

T.L. contributed to the overall concept, designed, performed, and analyzed experiments and wrote the manuscript. M.Z. designed, performed, and analyzed wire myography and rat experiments. S.H. contributed to pharmacokinetic experiments. R.J. and S.W. contributed to confocal microscopy. L.Z. contributed to EPR experiments. G.Z. contributed to HPLC‐MS^2^ experiments. H.S. and Q.L. contributed to data interpretation and manuscript preparation. A.B.B. contributed to the overall concept, experimental design, and manuscript preparation.

## Supporting information

Supporting Information

## Data Availability

The data that support the findings of this study are available in the supplementary material of this article.
