# Supplementary material for: H2S Increases Blood Pressure via Activation of L‐Type Calcium Channels with Mediation by HS• Generated from Reactions with Oxyhemoglobin
Source: Adv Sci (Weinh). 2024 Apr 29;11(26):2305866. doi: 10.1002/advs.202305866 (PMC11234399; doi:10.1002/advs.202305866)
Supplement: Supplementary file 1 — Supporting Information [file ADVS-11-2305866-s001.pdf]

ADVANCED SCIENCE
Open Access

Supporting Information

for *Adv. Sci.*, DOI 10.1002/advs.202305866

H_2S Increases Blood Pressure via Activation of L-Type Calcium Channels with Mediation by $HS^\bullet$ Generated from Reactions with Oxyhemoglobin

*Taiming Liu, Meijuan Zhang, Shawn Hanson, Rucha Juarez, Sean Wilson, Hobe Schroeder, Qian Li, Lingchao Zhu, Guangyu Zhang and Arlin B. Blood**

Supplementary Materials

H_2S increases blood pressure via L-type calcium channel-dependent vasoconstriction with mediation by $HS^•$ radicals generated from reactions with oxyhemoglobin

Taiming Liu[1], Meijuan Zhang[1], Shawn Hanson[2], Rucha Juarez[2], Sean Wilson[2], Hobe Schroeder[2], Qian Li[3], Lingchao Zhu[4], Guangyu Zhang[5], Arlin B. Blood[2*]

[1]Division of Neonatology, Department of Pediatrics, Loma Linda University School of Medicine, Loma Linda, CA 92354;
[2]Lawrence D. Longo Center for Perinatal Biology, Loma Linda University School of Medicine, Loma Linda, CA 92354;
[3]Neonatal Redox Biology Laboratory, Division of Neonatology, University of Alabama at Birmingham, Birmingham, AL 35294;
[4]Department of Chemistry, University of California, Riverside, CA, 92521;
[5]Mass spectrometry core facility, Loma Linda University, Loma Linda, CA 92354.

**Address Correspondence to:*
Arlin B. Blood
11175 Campus Street, 11121 Coleman
Loma Linda, CA 92354
Phone: 909-558-7448
Fax: 909-558-0298
Email: ablood@llu.edu

Preparation of S-nitroso-glutathione. S-nitroso-glutathione (GSNO) was prepared by incubating glutathione with an equimolar amount of sodium nitrite in 0.5 M HCl for 30 min on ice. The stock solution was pH-neutralized with NaOH and diluted immediately before use with HEPES buffer at pH = 7.4 containing 0.1 mM diethylene triamine pentaacetic acid (DTPA; a metal chelator).

H_2S measurements by HPLC-MS^2. Plasma H_2S concentrations were measured by HPLC-ESI-MS^2 similar to a previous report [28]. Briefly, 50 µl plasma was mixed with 200 µl of methanol solution containing 5 mM HPE-IAM (derivatization reagent; Supplementary Fig. 1) and 5 µM ^{34}S-labeled Na_2S (internal standard), and derivatized at 37 °C for 30 minutes. The supernatant

was collected for measurement after deproteinization via centrifugation. The standard curve samples (0.1-100 μM) of Na_2S were made with a deoxygenated HEPES buffer.

Chromatography separation was performed in an Agilent 1200HPLC, equipped with a HILIC column (3 mm, 2.1×150 mm, Acclaim). Gradient mobile phases (A: 0.1% formic acid in water; B: 0.1% formic acid in methanol; 0.2 ml/min) were used for elution: 10% A maintained for 2 min, linearly increased to 99% over 10 min, then linearly decreased to 10% over 4 min, and maintained at 10% for another 3 min. Mass spectrometry was performed using an Agilent triple quadrupole mass spectrometer under positive mode using a collision energy of 20 eV. ^{32}S-labeled $H_2S/HS^-/S^{2-}$ was measured using a multiple reaction monitoring (MRM) transition of 389->121, whereas ^{34}S-labeled $H_2S/HS^-/S^{2-}$ was measured using a MRM transition of 391->121 (Supplementary Fig. 1c-d). The lower limit of quantification for this methodology is 100 nM.

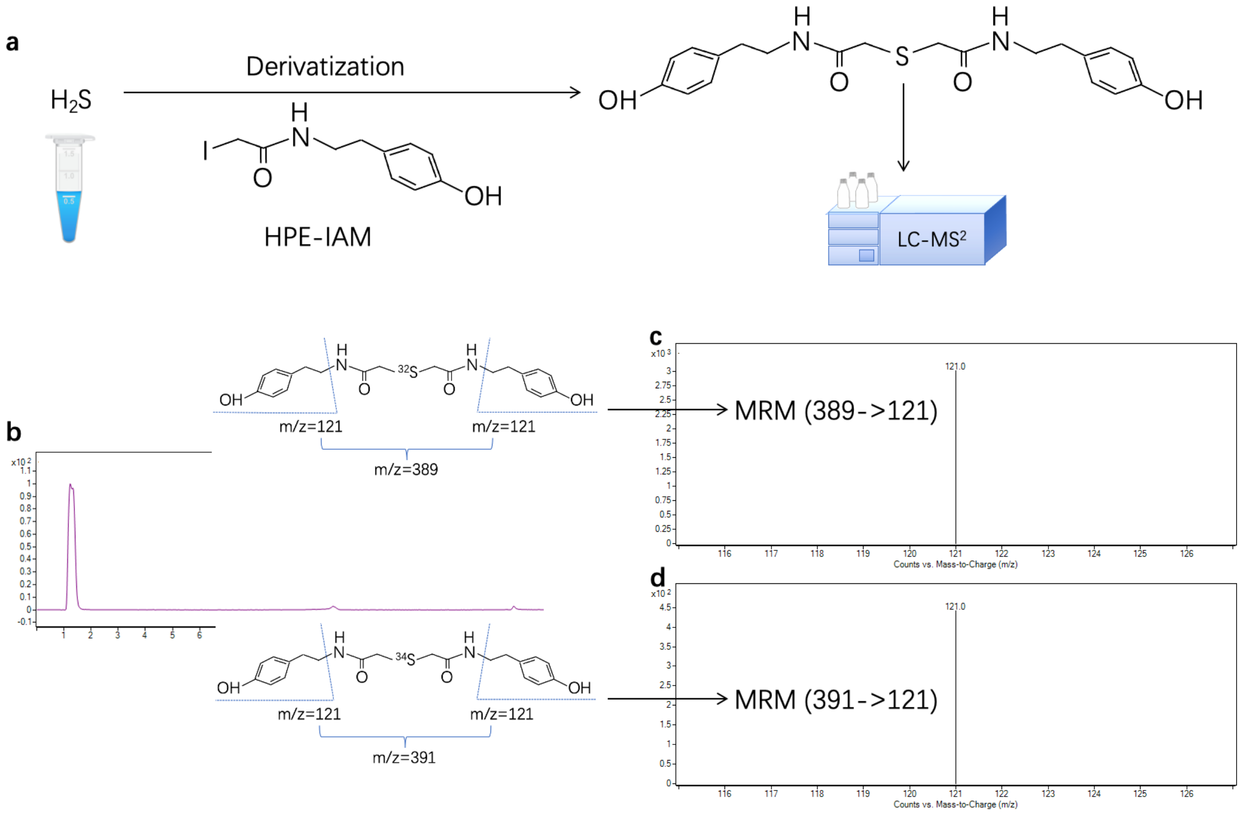

Supplementary Figure S1. H_2S measurements by HPLC-MS^2. **a)** Diagram for H_2S measurements by HPLC-MS^2. **b)** Liquid chromatography spectrum of bis(HPE-IAM)-$^{32/34}S$ adducts. **c)** MRM measurement of bis(HPE-IAM)-^{32}S adducts. **d)** MRM measurement of bis(HPE-IAM)-^{34}S adducts.

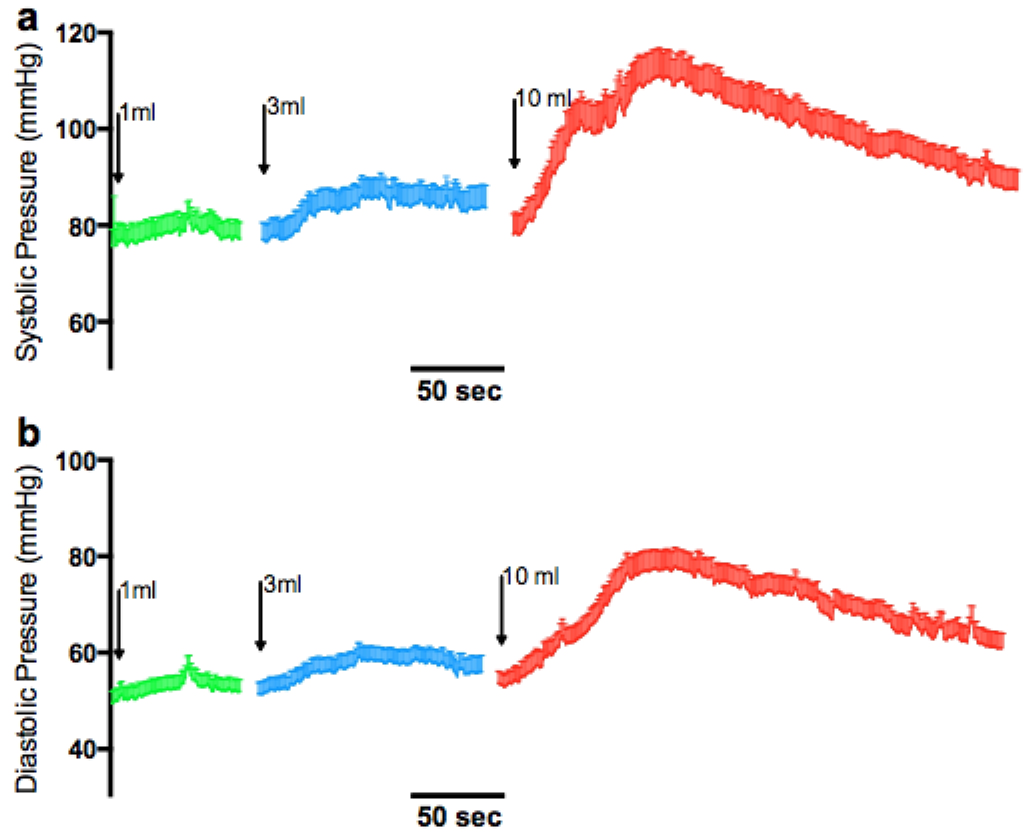

Supplementary Figure S2. Systolic **(a)** and diastolic **(b)** pressure responses of adult sheep to Na_2S injections. Na_2S (128 mM) boli were injected as 1, 3, and 10 ml boluses consecutively with an interval of 3 min. n = 32.

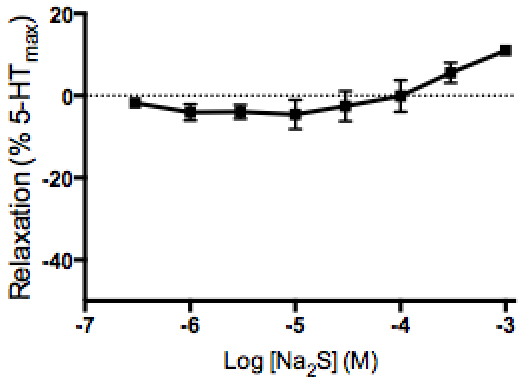

Supplementary Figure S3. Na_2S did not relax isolated sheep femoral arteries. Arteries were pre-constricted in the same manner of mesenteric arteries with 10 µM 5-HT. n = 3.

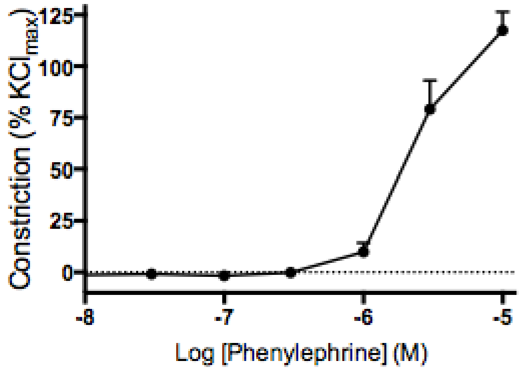

Supplementary Figure S4. Dose response curve of phenylephrine in isolated sheep mesenteric arteries. Data were normalized to the maximum contraction to 125 mM KCl (KCl_{max}) of the same vessel. n = 5.

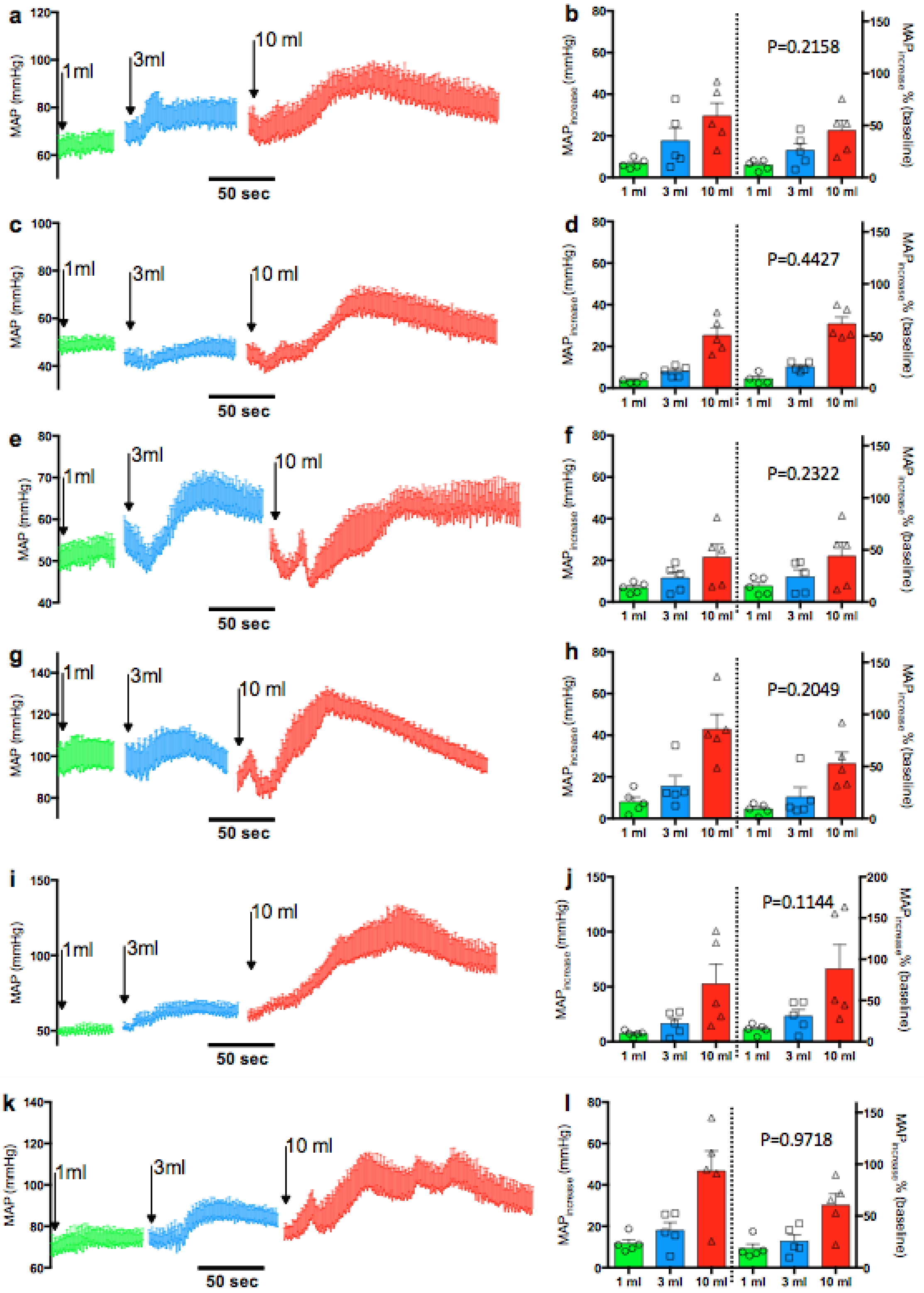

Supplementary Figure S5. Exclusion of the roles of carotid chemoreceptors, ganglionic activity, alpha-1 adrenergic activity, prostaglandins & COX1/2, cAMP pathway, and K_{ATP} channel on the hypertensive effects of H_2S in sheep. **a, c, e, g, i, k)** Averaged traces showing dose-dependent

increases of mean arterial blood pressure (MAP) by 128 mM Na_2S injections. **b, d, f, h, j, l)** Absolute (left) and relative (right) increases of MAP. **a-b)** Carotid chemoreceptor denervation, **c-d)** ganglionic activity blocker hexamethonium, **e-f)** alpha-1 adrenergic activity blocker prazosin, **g-h** prostaglandin and COX1/2 inhibitor indomethacin, **i-j)** cAMP pathway agonist isoproterenol, and **k-l)** K_{ATP} blocker glibenclamide did not significantly alter the hypertensive effects of H_2S. p value represents the results of 2-way ANOVA of relative MAP increase versus intact sheep (shown in Figure 1**b right** of the main manuscript).

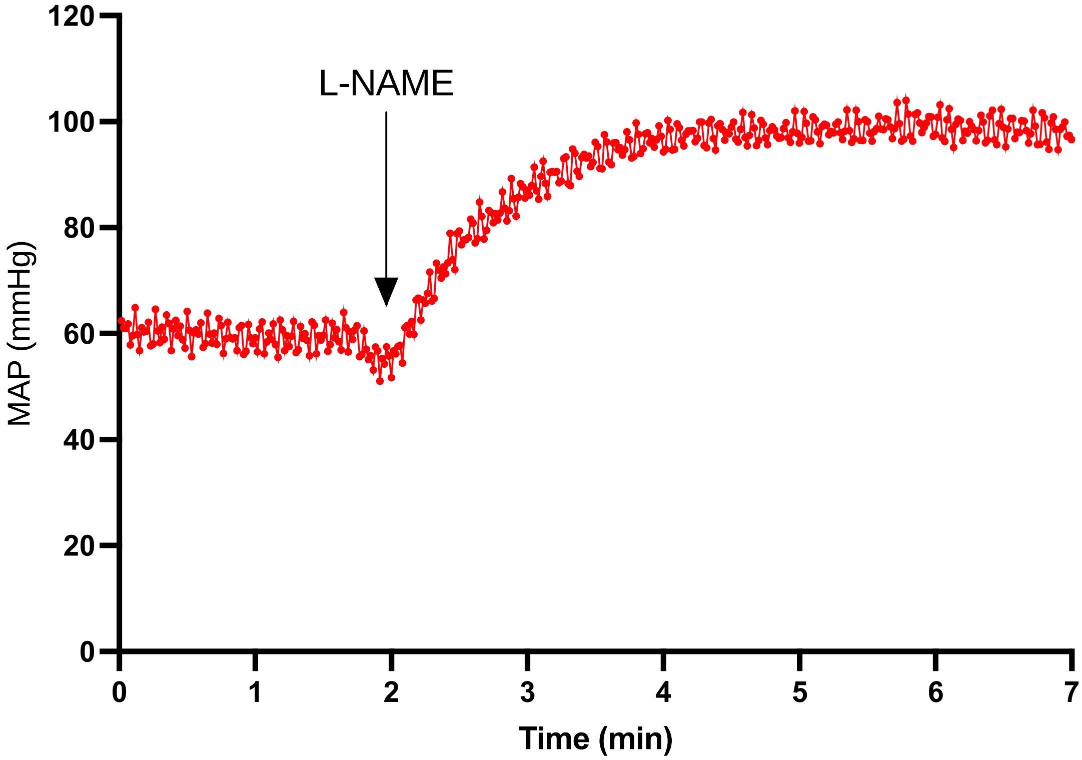

Supplementary Figure S6. Rapid effects of L-NAME on mean arterial blood pressure. Representative trace of MAP from one sheep.

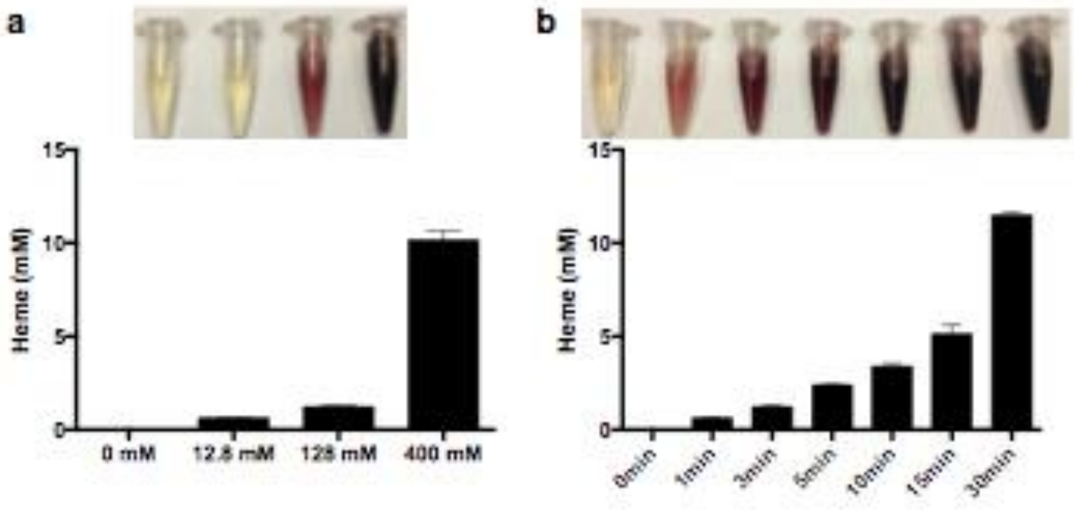

Supplementary Figure S7. Na_2S causes hemolysis. *n* = 3. **a** Dose dependence of Na_2S at 3 min. **b)** Time dependence of Na_2S at 128 mM. Blood was incubated with Na_2S at 1:1 volume ratio, and centrifuged at 12,000 rpm for 1 min. Heme concentrations were measured in the supernatant fractions and then adjusted to the dilution factor of 2. Representative pictures of the supernatant fractions were shown above the bar graphs.

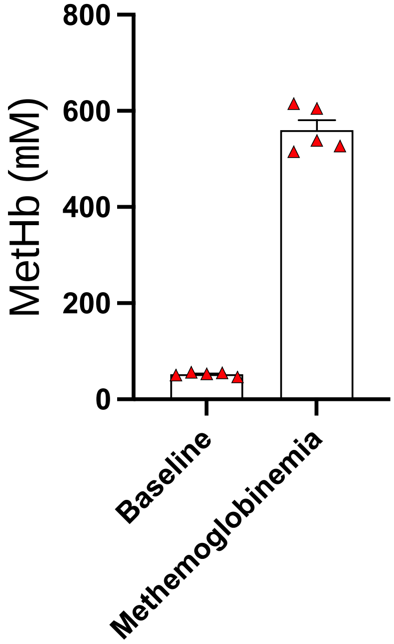

Supplementary Figure S8. Circulatory metHb levels in sheep at baseline and after introduction of methemoglobinemia. n=5. MetHb level was measured using Drabkin's reagents. Briefly, methemoglobin reacts with cyanide to generate cyanmethemoglobin that is quantified through colorimetry at 540 nm.

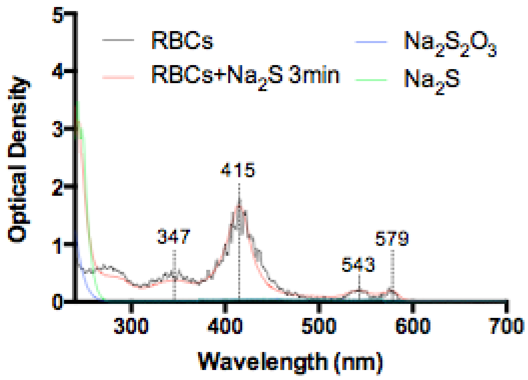

Supplementary Figure S9. UV-VIS absorption spectra showing that Na_2S does not alter UV-VIS absorption characteristics RBCs. *n* = 3. The spectrum of RBCs + Na_2S was obtained after the incubation of 3 ml of 128 mM Na_2S and 3 ml of RBCs at room temperature for 3 min in a beaker open to air. Spectra of RBCs, thiosulfate ($Na_2S_2O_3$), and Na_2S alone are also shown.

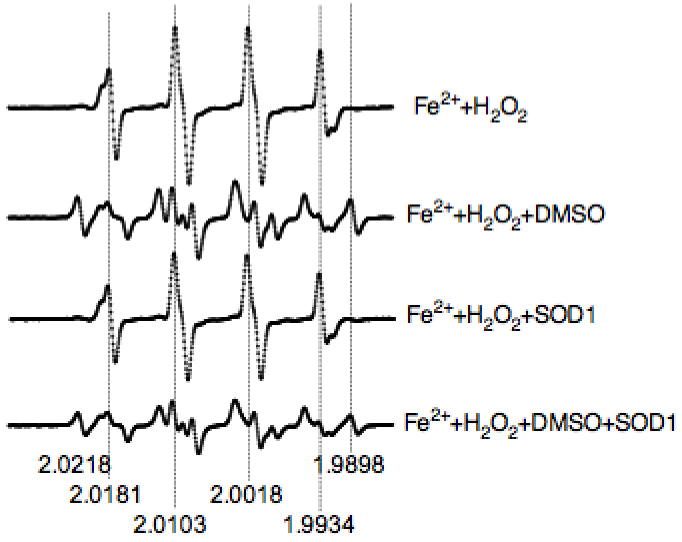

Supplementary Figure S10. Representative EPR traces demonstrating effective scavenging of hydroxyl and superoxide by DMSO and SOD1, respectively. BMPO (25 mM), which detects HS•, OH•, $O_2^{\bullet-}$, and carbon-centered radicals, was used as the spin trap for all measurements. Fenton reagents (10 mM $FeSO_4$+10 mM H_2O_2) were applied to generate OH• and $O_2^{\bullet-}$ to obtain BMPO-OH and BMPO-OOH, while DMSO (500 mM) and SOD1 (1000 U/ml) were used as

OH• and $O_2^{•-}$ scavengers, respectively. All traces were shown under the same scale and aligned with g factor (numbers at the bottom). The above peaks were not observed in our studies of HS•, indicating successful elimination of OH• and $O_2^{•-}$ radicals for the HS• detection. *n* = 3.

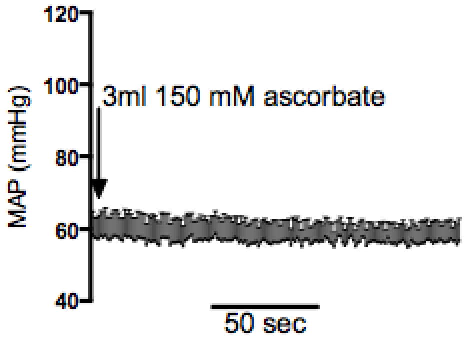

Supplementary Figure S11. Sodium ascorbate (3 ml bolus of 150 mM) injected alone did not alter blood pressure. *n* = 3.

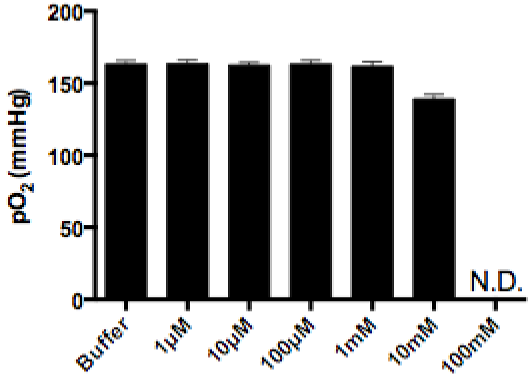

Supplementary Figure S12. Na_2S at millimolar levels decreases the partial pressure of O_2 (pO_2) in Hepes buffer (containing 0.1 mM DTPA) in a concentration dependent manner. *n* = 3.

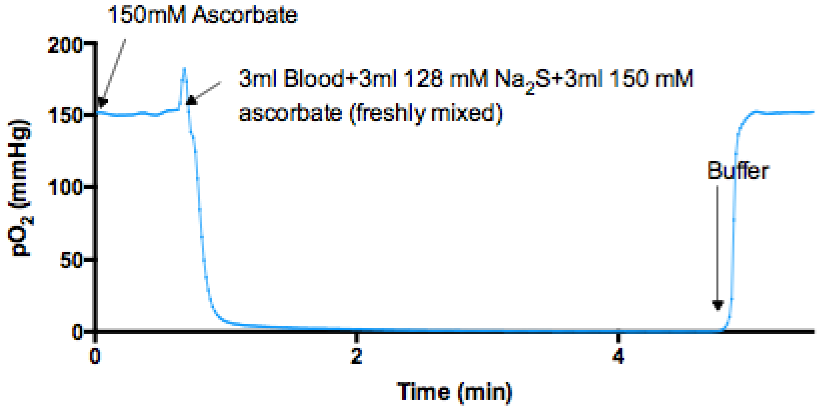

Supplementary Figure S13. Na_2S decreases blood pO_2 in the presence of sodium ascorbate. A fluorometric O_2 probe was successively inserted into different solutions for measurements of pO_2. Three ml of fresh sheep blood was mixed with 3 ml of 128 mM Na_2S and 3 ml 150 mM

ascorbate in an air tight syringe, while HEPES buffer was exposed to air as a positive control.

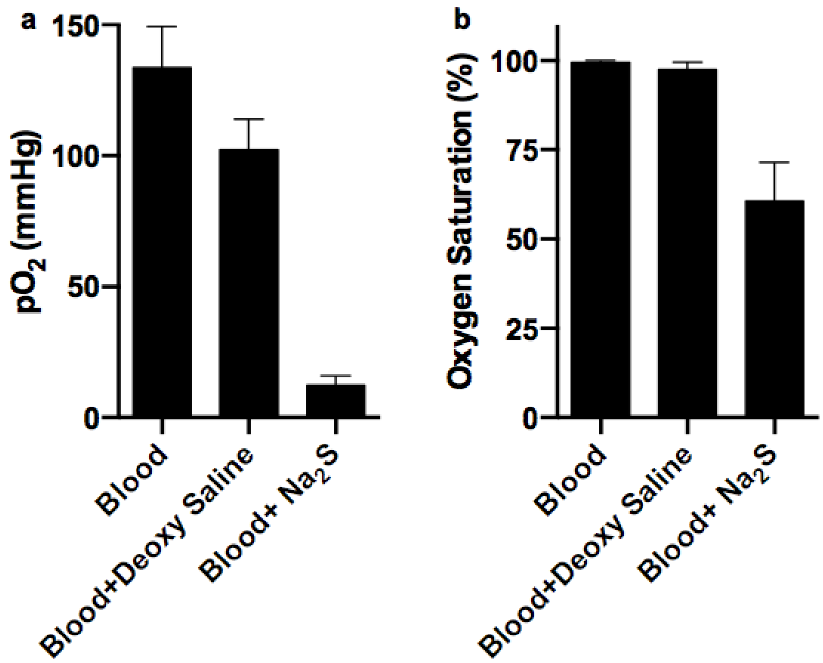

Supplementary Figure S14. Na_2S decreases blood pO_2 (**a**) and oxygen saturation (**b**) as measured by a blood gas analyzer which utilizes amperometric and spectrophotometric methods. n = 3. Three ml of 128 mM Na_2S was incubated with 3 ml arterial blood in a sealed syringe for 3 min before measurements. Because Na_2S eliminates O_2 in buffer, saline deoxygenated by equilibraion with 100% N_2 was used as the control for 128 mM Na_2S.

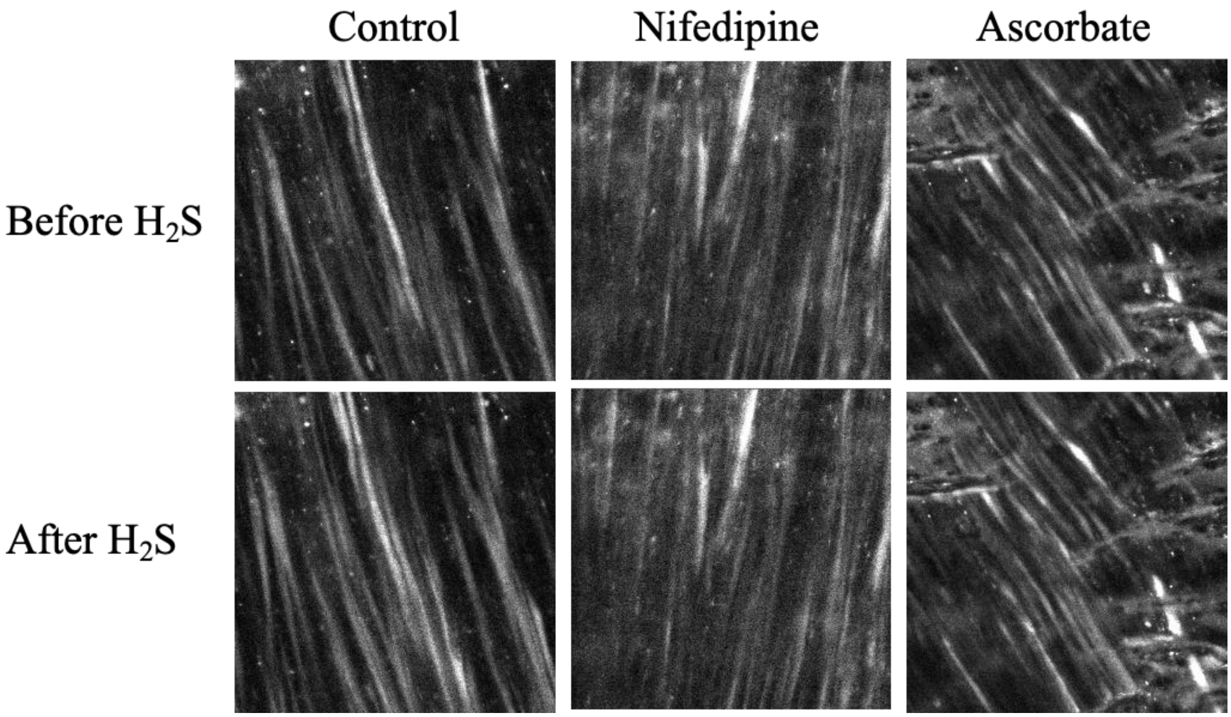

Supplementary Figure S15. Representative images of the confocal microscopy measurements of Ca^{2+} signal in isolated sheep mesenteric arteries. Nifedipine and ascorbate blocked the Na_2S-induced increase in Ca^{2+} signal. Images came from three endothelium-denuded arterial segments from one sheep.

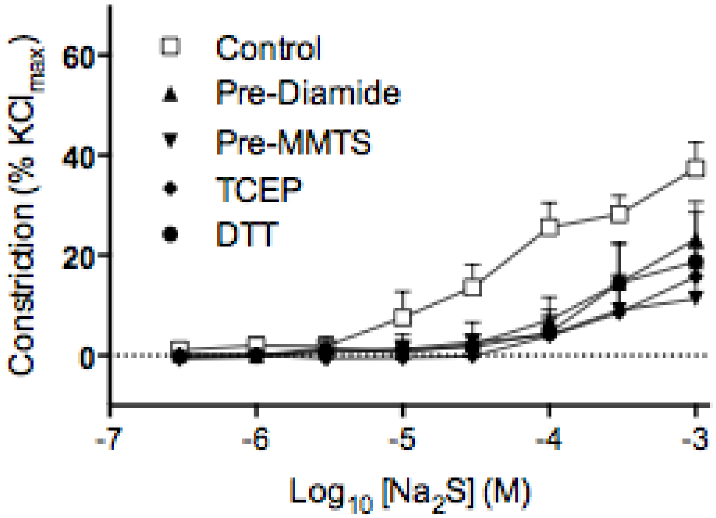

Supplementary Figure S16. Evidence for involvement of redox-sensitive thiol(s) in the vasoconstriction induced by H_2S. Na_2S-induced vasoconstriction was inhibited in the presence of disulfide bond breakers TCEP and DTT and by the pretreatment (pre-) of the isolated arteries with thiol oxidizers diamide and MMTS. Experiments were performed in the presence of 1 μM phenylephrine and 2% v/v RBCs. Data were normalized to the maximum contractile response to 125 mM KCl (KCl_{max}) of the same vessel. $n \geq 4$.

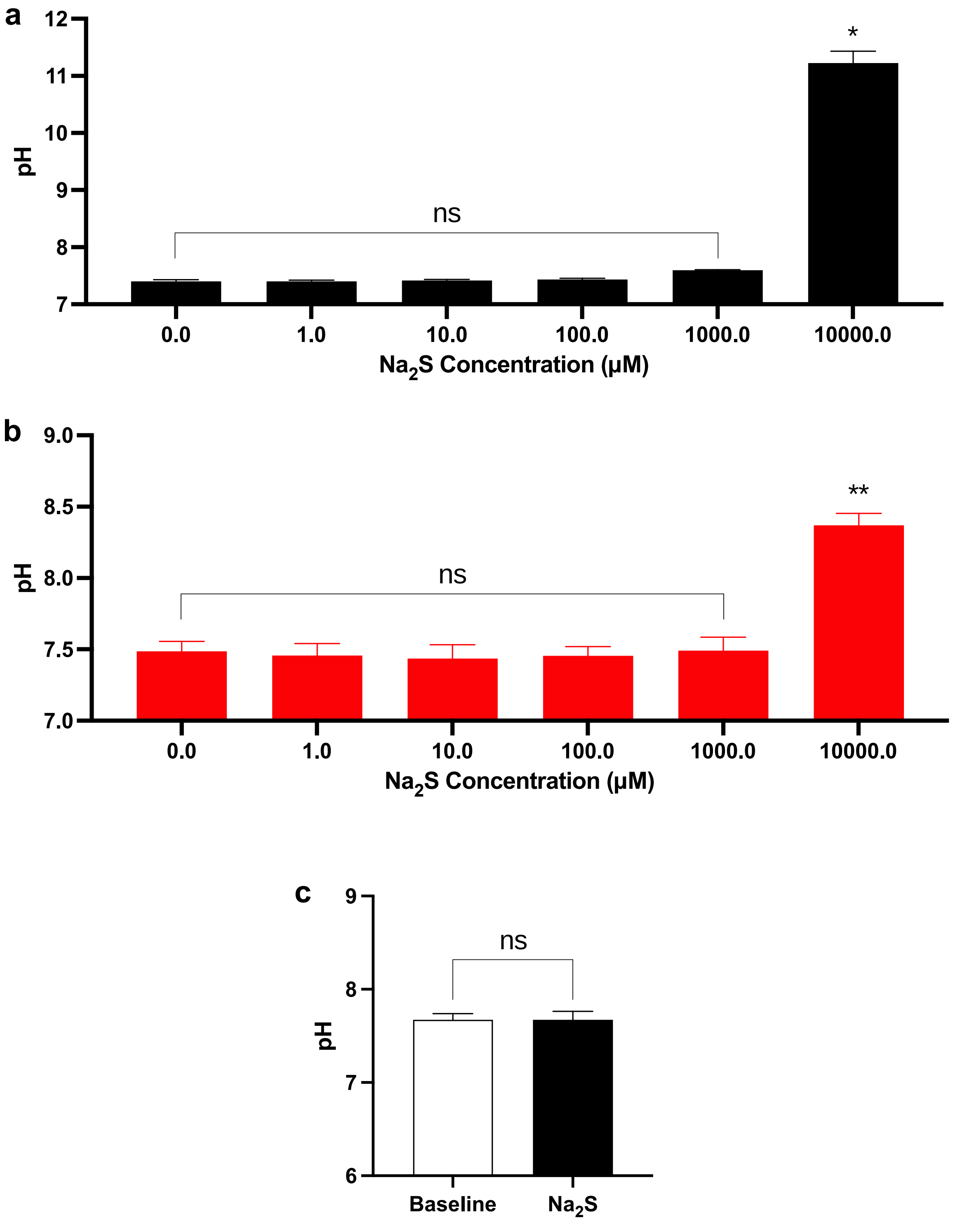

Supplementary Figure S17. Effects of Na_2S on pH. Addition of Na_2S to concentrations as high as 1 mM does not significantly alter pH in HEPES buffer (**a**; 10 mM, pH=7.40) or sheep whole blood (**b**) as measured in vitro by pH meter. n=3. One-way ANOVA with Sidak's test. (**c**) Bolus injections of Na_2S (10 ml of 128 mM Na_2S) to sheep do not significantly alter pH in arterial blood as measured by a blood gas analyzer. n=8. Two-tailed paired t-test.

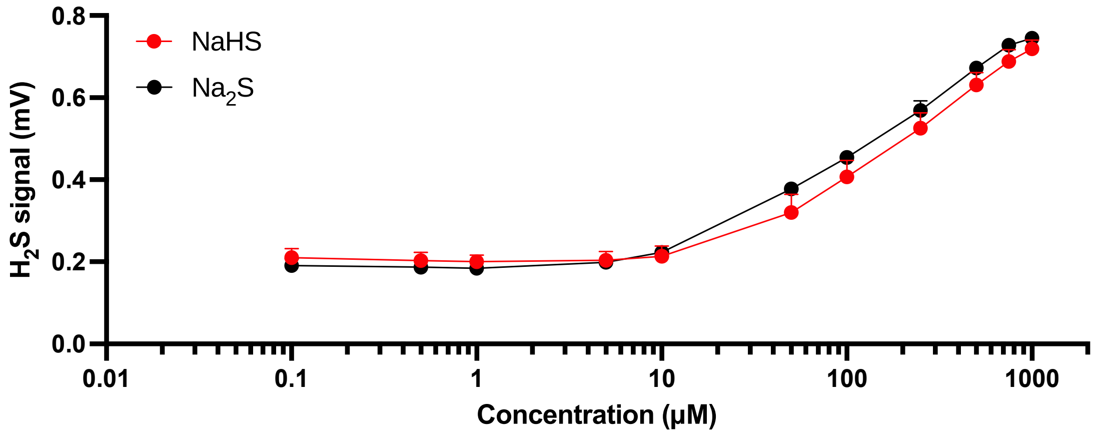

Supplementary Figure S18. Parallel comparison of H_2S release from Na_2S and NaHS in HEPES buffer. n=3. H_2S-specific amperometric electrode was inserted into the headspace of a sealed flask (50 ml) containing 30 ml of continuously-stirred HEPES buffer. Na_2S and NaHS were cumulatively injected into the HEPES buffer through a catheter placed at the bottom of the flask. As shown, Na_2S and NaHS have similar ($p=0.5514$; Two-way ANOVA) H_2S releasing properties.

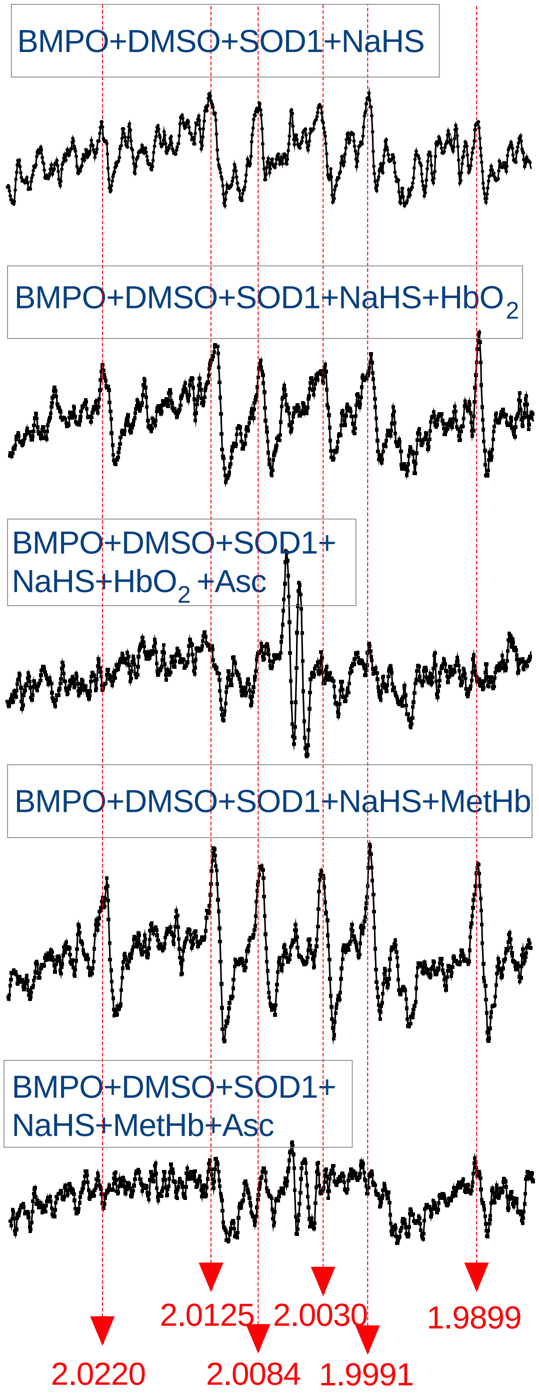

Supplementary Figure S19. NaHS generates HS•. n=3. Representative EPR spectrum. Like Na_2S, NaHS also generates HS• by reaction with HbO_2 and metHb. The HS• is scavenged by radical scavenger sodium ascorbate.
